# Lessons learned from Evidence-Informed Decision-Making in Nutrition & Health (EVIDENT) in Africa: a project evaluation

**DOI:** 10.1186/s12961-019-0413-6

**Published:** 2019-01-31

**Authors:** Pankti Motani, Anais Van de Walle, Richmond Aryeetey, Roosmarijn Verstraeten

**Affiliations:** 1 Department of Public Health, Institute of Tropical Medicine, Antwerp, Belgium; 2 Department of Population, Family and Reproductive Health, University of Ghana, Accra, Ghana; 3 Independent Researcher, Antwerp, Belgium

**Keywords:** Evidence-informed decision-making, low- and middle-income countries, evaluation, lessons learned, capacity-building, leadership, stakeholder engagement, contextualisation, network

## Abstract

**Background:**

Evidence-informed Decision-making in Nutrition and Health (EVIDENT) is an international partnership that seeks to identify information needs in nutrition and health in Africa and build local capacity in knowledge management to help translate the best available evidence into context-appropriate recommendations aligned to the priorities of decision-makers. This study evaluates the extent to which EVIDENT achieved its intended activities, documents the lessons learned and draws on these lessons learned to inform future activities of EVIDENT, as well as in evidence-informed decision-making (EIDM) in nutrition overall.

**Methods:**

Purposive and snowball sampling were used to identify participants that were either directly or indirectly involved with EVIDENT. An analytical framework of five key elements was developed to guide data collection from EVIDENT’s documentation, in-depth interviews (*n =* 20), online surveys (*n* = 26) and a participatory discussion. Interviews were transcribed verbatim and coded in NVivo 11, using deductive thematic content analysis and a phenomenological approach. Online surveys were analysed using Stata 14. Data were triangulated to address both objectives under each element of the analytical framework.

**Results:**

EVIDENT succeeded in establishing a collaborative partnership, within which it delivered four short courses in EIDM. This capacity complemented case study activities in four partner African countries where EIDM processes were implemented and assessed. Identified barriers to these processes included little experience in EIDM, difficulties in engaging stakeholders, challenging local environments (e.g. donor influence, bureaucracy, inaccessibility to scientific research, poor internet connectivity), and limited time and funding. However, EVIDENT activities were driven by a local need for EIDM, a sheer interest and commitment to the cause, and the opportunity for the Global North and South to work together and build relationships. Future activities of EVIDENT, and EIDM in nutrition overall, should focus on sustained capacity-building in EIDM processes, leadership and functional skills across the Global South, investment in stakeholder engagement, context-specific EIDM, enhanced communication and linking, and strengthening relationships with existing stakeholder organisations.

**Conclusions:**

In its first 3 years, EVIDENT developed and strengthened partnership, capacity and visibility on EIDM in Africa. Innovative and long-term capacity-building, dedicated leadership, further stakeholder engagement and sustainable financing, are needed for future activities of EVIDENT and EIDM in nutrition.

**Electronic supplementary material:**

The online version of this article (10.1186/s12961-019-0413-6) contains supplementary material, which is available to authorized users.

## Background

Over the past years, evidence-informed decision-making (EIDM) and policy-driven nutrition research have gained traction in advancing public health globally, and more recently in low- and middle-income countries (LMICs) [1, 2]. The need to make the best use of available resources, combined with the urge to progress in addressing the malnutrition burden [3], has fuelled the growing demand to justify decision-making at every level in LMICs. A multiplicity of factors influences the process of making decisions. In high-income countries (HICs), some of the factors that influence public health policy-making include interactions between researchers and decision-makers, mutual trust, timeliness, under-resourcing, power and budget struggles, and the inclusion of summaries with policy recommendations [4–6]. Applying EIDM in LMICs, however, comes with additional challenges [7, 8]. Barriers and practical constraints that need to be overcome to use research evidence in decision-making in LMICs often include poor quality and inaccessibility of research, a paucity of locally relevant research, little or no funding, and a lack of addressing the decision-maker’s information needs. These barriers are further accompanied by insufficient (institutional) capacity to identify and use the best available evidence as well as a lack of leadership [7, 9]. Furthermore, to prioritise needs for decision-making, the “*relevance, applicability or generalisability*” of the evidence to a specific context is crucial [2]. In addition, while evidence on its own is important, it is an insufficient driver of a good decision-making process. Other factors such as economic constraints, local context (administrative and governance traditions, and values), and ethical constraints also play a critical role in the decision outcome [7, 10]. The need to enhance technical capacity and leadership skills at all stages of the EIDM process to obtain better decision outcomes in LMICs has been re-iterated in the literature [11, 12].

In response to the identified gaps in capacity, a number of initiatives have emerged to strengthen leadership and capacity in EIDM for nutrition and health. Examples of initiatives focusing on agriculture and food policies in improving nutrition and health outcomes include Agriculture for Nutrition and Health Research, Leveraging Agriculture for Nutrition in South Asia and Leveraging Agriculture for Nutrition in East Africa [13–15]. Others, like the VakaYiko Consortium, aim to strengthen the capacity of policy-makers by developing an evidence-informed policy-making toolkit to support the use of evidence in policy-making across sectors in Africa [16–18]. The Africa Evidence Network links researchers, practitioners and policy-makers working in research, civil society and government agencies across Africa to promote evidence production and use in decision-making [19]. The network provides opportunities to share technical capacities in EIDM within and outside Africa. Holdsworth et al. [8] have provided a detailed overview of different EIDM initiatives in nutrition. Despite these initiatives, capacity and leadership to generate and synthesise evidence to inform policy and programming decisions remains limited, particularly in the African context. Where evidence is generated, it is mostly done by external academic institutions with limited involvement of local, national and sub-national priorities [17]; this is the case even in settings where priorities have already been identified by governments [20]. Meanwhile, existing processes of decision-making in African settings have continued to remain unexplored and undocumented for overall lesson learning towards EIDM [7, 9].

It is against this background, and in response to the expressed concerns of African stakeholders, that the Evidence-informed Decision-making in Nutrition and Health (EVIDENT) partnership emerged. EVIDENT is an international partnership that seeks to strengthen capacity in addressing the disparity between research activities and local evidence needs in nutrition and health in Africa [21, 22]. Its activities include identifying local information needs at national or subnational levels and building capacity in knowledge management, and to translate the best available evidence into locally appropriate tangible recommendations that are aligned to the priorities of decision-makers. The ultimate goal is to enable and promote decisions informed by best evidence within the boundaries allowed by local context. EVIDENT’s objectives and activities are described elsewhere in detail and summarised in Box 1 [8, 21].

The aim of this study is to evaluate EVIDENT’s progress in its goals and objectives, to assess the achievements in terms of added value and quality of the project, to identify the lessons learned on EIDM such as drivers, barriers and opportunities, and to draw on these lessons learned, in the first 3 years (2013–2016) of implementation. This evaluation was also carried out to meet regular reporting requirements in accordance with commitments to the funding agency. We specifically aimed to (1) assess the extent to which EVIDENT’s intended activities were implemented and to document the lessons learned (drivers, barriers and opportunities) from the first 3 years of this project (proving), and (2) draw on the lessons learned to inform future activities of EVIDENT and EIDM in nutrition overall (improving).

## Methods

### Ethical considerations

Ethical approval for this evaluation was obtained from the Institutional Review Board (1112/16) at the Institute of Tropical Medicine, Belgium. Signed written consent forms were completed by all participants. Verbal consent was also obtained from each participant prior to recording their interview. Each participant was given a unique identifier to ensure that all responses were treated anonymously and confidentially. The Consolidated Criteria for Reporting Qualitative Research (COREQ): a 32-Item Checklist for Interviews and Focus Groups were used to report the results (Additional file 1) [23].

### Analytical framework

**Fig. 1:**
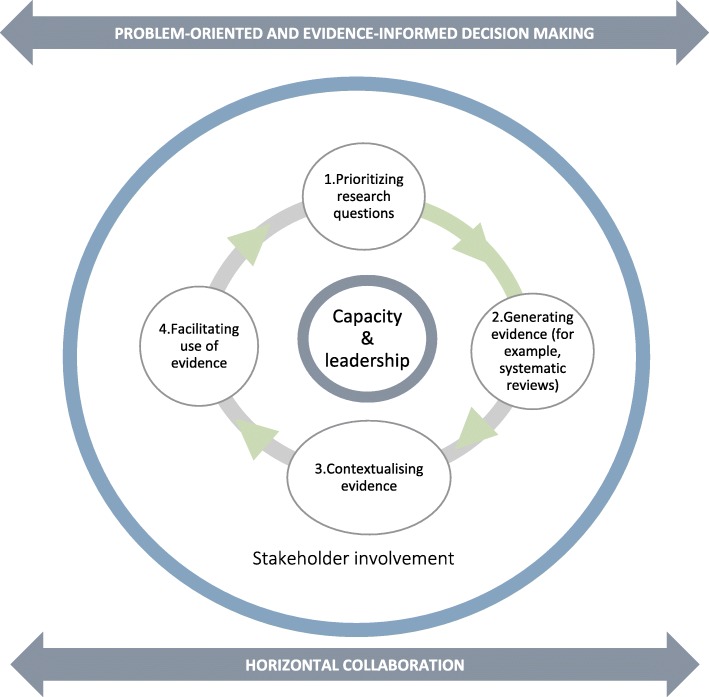
EVIDENT’s conceptual framework for evidence-informed decision-making

The three pillars of EVIDENT’s conceptual framework (Fig. 1) formed the core thematic elements by which activities within EVIDENT were evaluated. Two additional elements were identified from literature related to enabling environments for EIDM [1, 2] and from discussions within the research team [12, 24]. The identified elements were interconnected and complementary to each other, and their relative importance to EIDM and changeability (i.e. how feasible it was to change the elements) were evaluated prior to inclusion in the analytical framework. For each of the elements, lessons learned (drivers, barriers, opportunities) and unintended consequences were extracted. Together, the elements formed the analytical framework used for this evaluation (Table 1). This framework guided the collection, summary and reduction of the quantitative and qualitative data. The elements of the analytical framework also underpinned the semi-structured in-depth interviews, the online surveys and documentation review, and were aligned for analysis across these three instruments.

**Table 1:** Analytical framework of key elements

Elements	Source
Capacity-building	CF
Governance and leadership	SL/CF
POEIDM – case studies	CF
Horizontal collaboration	CF
Network and communication	CF
Visibility	CF
Sustainability	SL/RT

*CF* conceptual framework, *POEIDM* problem-oriented and evidence-informed decision-making, *RT* research team, *SL* scientific literature

### Participants

Participants invited to take part in this evaluation included EVIDENT partners (i.e. directly involved actors; groups I and II) as well as key stakeholders associated or linked with EVIDENT (i.e. indirectly involved actors; group III) (Table 2). Participants were identified by the evaluators (PM and AVDW) via purposive sampling and using EVIDENT’s documents such as meeting reports, protocols and the project website. Snowball sampling was used to identify any additional actors indirectly involved with EVIDENT (group III).

**Table 2:** Group criteria of invited participants

Level of involvement	Group (*n*)	Participant classification criteria	Participant institutions/organisations	Data collection instruments
Directly involved	I (17)	EVIDENT partners, who were actively involved with the development of EVIDENT’s framework, its core activities and case studies	− Ethiopian Public Health Institute (EPHI), Ethiopia − Institute of Tropical Medicine (ITM), Belgium − National School of Public Health, Morocco − North-West University (NWU), South Africa − Royal Tropical Institute, the Netherlands − Sokoine University of Agriculture, Tanzania − University of Abomey-Calavi, Benin − University of Ghana, Ghana − University of Ghent, Belgium − University of Sheffield, UK	− In-depth interview − Online survey
	II (7)	Actors who are/were involved for some period of time (1 or 2 years) in the development of EVIDENT’s framework, its core activities and case studies	− EPHI, Ethiopia − Federal Knowledge Centre (KCE), Belgium − International Food Policy Research Institute (IFPRI) − NICE International, UK − University of Sheffield, UK	− In-depth interview − Online survey
Indirectly involved	III (40)	Actors who are/were indirectly involved with EVIDENT; these include, for example, EVIDENT’s training participants, external collaborators at conference presentations or funding agency representatives	− Centre for evidence-based healthcare, South Africa − Charlotte Maxeke Research Consortium, South Africa − Cochrane, South Africa − General Secretary of Health, Morocco − Health Intervention and Technology Assessment Program, Thailand − International Network of Agencies for Health Technology Assessment, Canada − ITM, Belgium − KCE, Belgium − Makerere University, Uganda − Micronutrient Initiative, Canada − Ministry of Health, Ethiopia − NICE International, UK − NWU, South Africa − Priority Cost Effective Lessons for Systems Strengthening, South Africa − Scaling Up Nutrition (SUN) Movement − Stellenbosch University, South Africa − University of Abomey-Calavi, Benin − University of Cape Coast, Ghana − University of Ghana, Ghana − University of Ghent, Belgium − University of Sheffield, UK	− (Shorter) Online survey

### Data collection instruments

Both quantitative (online surveys and documentation) and qualitative data collection instruments (in-depth interviews) were used during the evaluation.

#### Documentation

A variety of EVIDENT’s documents (e.g. reports, website, brochures) amassed over the 3-year duration of the partnership were reviewed and used for data extraction. Data were summarised into Microsoft Word (2010) according to the structure of the analytical framework. Where needed, EVIDENT’s partners and stakeholders were contacted to provide any further information or clarifications.

#### In-depth interviews

Individuals directly involved with EVIDENT (groups I and II; *n* = 24) were invited to take part in the in-depth interviews. The interviews were audio-recorded, led by an interviewer (AWDV) and attended by a silent observer (PM) who noted non-verbal individual behaviour such as reluctance to answering a certain question, sarcasm, confusion or laughter [25]. Interviews were conducted in English and over teleconference call software, Skype (https://www.skype.com/en/). When a Skype call was not possible, typically due to poor internet connection, interviews were carried out face-to-face, in person. A short debriefing was held between the interviewer and the observer after each interview to discuss any highlights or noteworthy points. Interviews lasted between 15 minutes and 1.5 hours. After each interview, participants were asked to identify additional individuals they felt could contribute to the EVIDENT project evaluation; this snowball sampling technique targeted those participants who were indirectly involved with EVIDENT (group III).

Using the analytical framework, a semi-structured questioning guide, adapted to the target group, was developed, pre-tested and refined (Additional file 2 and Additional file 3). The questions were designed to solicit information about the different key elements identified in the framework (Table 1). Open-ended questions were followed by more specific probes to clarify and elaborate responses where needed.

#### Online surveys

Two self-administered online surveys were used to collect quantitative data from the participants. Both surveys collected sociodemographic data. Twenty participants from groups I and II, those who took part in the interviews, were invited to complete the survey (Additional file 4). Participants from group III, identified through documentation and snowball sampling (*n* = 40), received a shorter survey asking participants’ overall views on EVIDENT (Additional file 5). The surveys were administered using online software, Survey Monkey (https://www.surveymonkey.com/), and comprised both closed- and open-ended questions. Response categories included 5-point Likert scales and pre-specified response categories.

### Recruitment

Each participant was formally invited to take part in the evaluation via email. This email provided detailed information on the nature of the study. Up to two email reminders were sent out to potential participants within 17 days of the initial invitation. If no response was registered after the second reminder, the individual was classified as a non-response. Once individuals accepted the invitation, they were asked to provide written informed consent. Groups I and II were asked to participate in both the in-depth interview and online survey; a web link to the survey was shared after the interview to avoid bias (Table 2). Group III received a web link to complete the shorter online self-administered survey (Table 2).

### Data coding and analyses

Recorded interviews were transcribed verbatim by PM, AVDW, and an external transcriptionist. All transcripts were verified by the two evaluators prior to their use in analyses. Deductive thematic content analysis and a phenomenological approach, which were based on the analytical framework of this study, were used [26]. Using this framework, both PM and AVDW independently read and coded five transcripts, to identify emergent themes.

Each identified theme, or ‘node’, was defined by the evaluators and definitions were discussed until an agreement was reached. In instances of no agreement, a third researcher was consulted (RV). Together, the nodes formed a node tree, which was then used to code all interview transcripts (Additional file 6). Both evaluators independently coded all transcripts. The codebooks were then agreed upon amongst the evaluators through discussions. NVivo software (QSR International – version 11.0, Melbourne, Victoria, Australia) was used to code and manage the data. Each node was assessed based on the frequency, specificity, emotion and extensiveness of the coded data [25], and then summarised into concise descriptions supported by relevant quotes and taking into account the non-verbal behaviour during analysis. In-depth interview attributes, such as partner country and setting, were cross-linked with themes across participants. Findings from the in-depth interviews, online surveys and documents were grouped into a matrix using the analytical framework (Additional file 7).

Sociodemographic and survey data were extracted, systematised and tabulated from Survey Monkey into Microsoft Excel (2010). These were summarised using Stata (Intercooled Stata version 14 Statacorp, College station, TX, USA), using frequencies and percentages. Where relevant, comments from open-ended questions in the online surveys were added to the summary of each node.

**Fig. 2:**
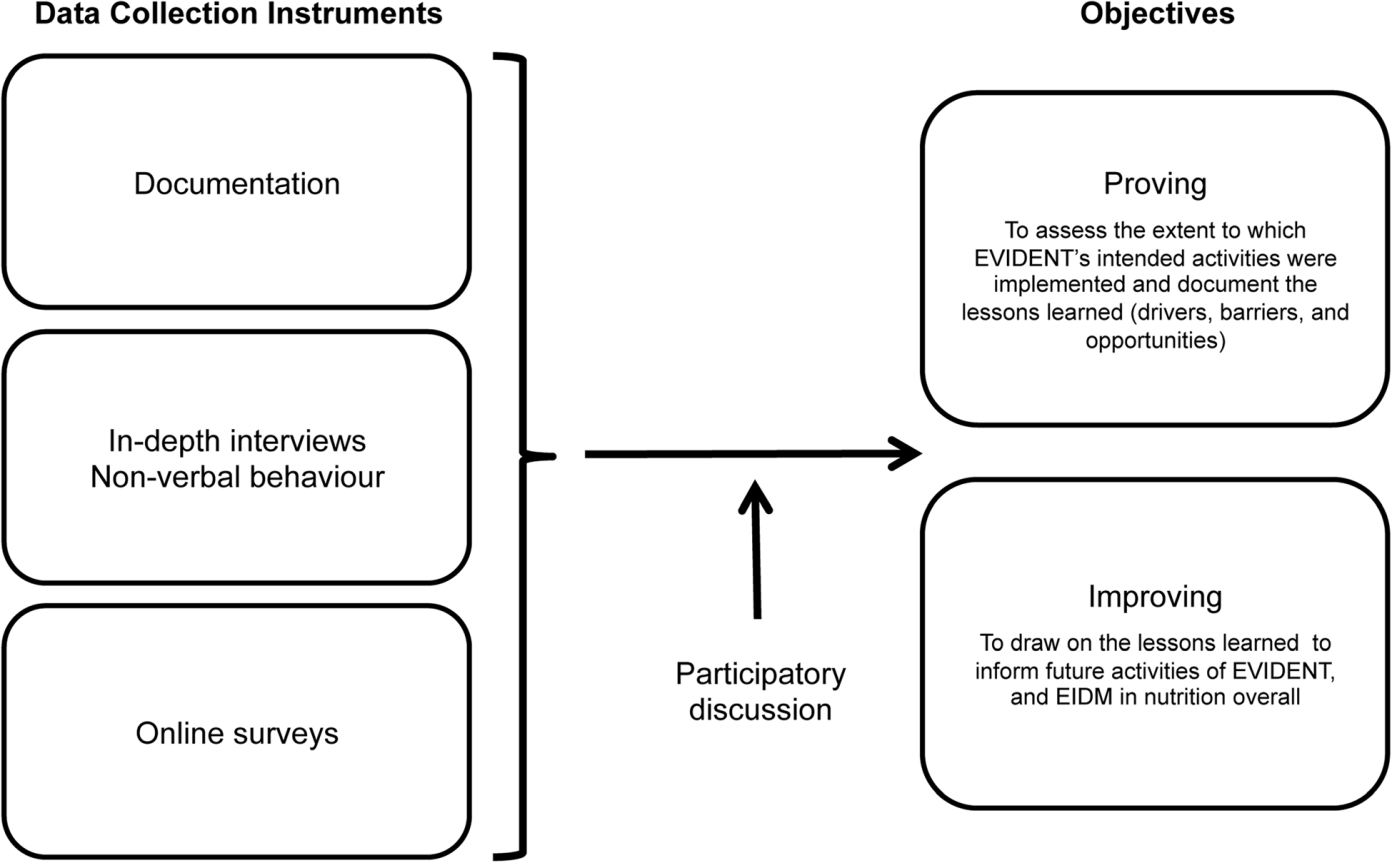
Contribution of various data collection instruments to the evaluation objectives

Evaluators used summarised coding from interviews, data from online surveys and EVIDENT documents to triangulate emerging themes from the collected data. Figure 2 illustrates how the various instruments of data were used to reach the objectives (proving and improving) of the project evaluation. Preliminary results from these data sources were presented at the final EVIDENT partner meeting (groups I and II) where participants were given the opportunity to express their views on the findings. Subsequently, the latter were integrated in the analysis of the evaluation to enhance the accuracy and understanding of the data collected [27].

## Results

Results are presented according to the five elements of the analytical framework to address the objectives of ‘proving’ and ‘improving’ EVIDENT (Table 1). Supporting data for these results have been grouped into a matrix (Additional file 7).

### Participants

Fifty-one individuals were identified from EVIDENT’s documents as potential participants; an additional 13 were identified through snowball sampling. Of the 64 individuals identified, 31 (49%) accepted the invitation to participate in this evaluation. In total, 20 in-depth interviews (16 in group I and 4 in group II) were conducted with participants directly involved in EVIDENT; 15 of these interviewees completed the online survey. Only 11 indirectly involved participants (group III) completed the online survey.

Those directly involved with EVIDENT were associated with the project for an average of 28.3 ± 7.8 months of the 36-month period; this is approximately 1.5 times as long as those who were indirectly involved (Table 3). Overall, 73% of directly involved participants and 45% of indirectly involved participants had expertise in nutrition. Other participants had expertise across medicine, international health, bioengineering sciences, qualitative synthesis, governance and development, economics, and political economics. All participants, except for two policy advisors, had professional backgrounds in research and academia.

**Table 3:** Evaluation participant characteristics

	Directly involved (groups I and II), *n* = 15	Indirectly involved (group III), *n* = 11
Length of involvement in EVIDENT (mean ± SD), months	28.26 ± 7.79	17.43 ± 14.42
Region of work, % (*n*)	− 66.66% Africa (10/15) − 33.33% Europe (5/15)	− 45.45% African (5/11) − 36.36% European (4/11) − 18.18% Asian (2/11)
Professional background in research and academia, % (*n*)	100%	81.81% (9/11)
Expertise in nutrition, %	73.33% (11/15)	45.45% (5/11)

### Proving

#### Capacity-building

EVIDENT delivered 4 short courses to a total of 67 researchers and policy-makers in 3 different countries; this represents 22 more individuals than the project originally intended to train. Two of the short courses took place in Belgium (*n* = 35), one in South Africa (*n* = 15), and one in Ethiopia (*n* = 17). Two face-to-face modules (‘systematic reviews’ and ‘translating evidence into country-specific recommendations and policy brief development’) were developed for these short courses, and each was delivered over a 2-week period. In addition, five virtual sessions were organised as follow-up sessions to enable experts in evidence synthesis (coaches from the scientific panel) to provide guidance to participants from short courses as they worked through the systematic review process.

As reflected in the responses from the online survey and interviews, the two modules were highly valued and had real benefits in terms of participants’ capacity-building. Interviewees felt that these modules provided insight into the importance of generating useful research evidence for EIDM and almost all reported that it taught them new skills. The perceived strengths of the short courses were the face-to-face approach combined with virtual follow-up, and content tailored to address the expressed needs of EVIDENT partners. The high value and need for such training was also reflected by the fact that an additional short course was organised in Benin and 18 additional individuals were trained.

In addition to the short courses, participants perceived EVIDENT as being successful in building different kinds of capacities such as engaging multi-sector partners, including donors and decision-makers, at local and international level, managing conflicts, exercising leadership in evidence synthesis activities (for both participants from the short courses and course coaches), and working in a multi-cultural context. Progress in utilising these competences was most successful when country teams established good relationships and communication with their coaches, which allowed for a mutual understanding of each other’s situation. Despite acquiring these competencies, insufficient time allocation to EVIDENT activities was frequently mentioned by interviewees as a limitation; participants were particularly constrained in incorporating the systematic reviews into their daily work schedule.

#### Governance and leadership

EVIDENT set up a management structure that was considered “*well-organised*” and satisfactory by most interviewees. This structure comprised of a coordinating body, country teams, a scientific expert panel of coaches and a quality assurance board. This extensive, yet organic and flexible structure, allowed tasks and responsibilities to be adaptable to partners’ requirements, which was perceived as a strength by several interviewees and 11 out of 16 online survey respondents. On the other hand, some interviewees thought this adaptive nature led to a lack of clarity in EVIDENT’s goals, objectives and deliverables. Moreover, some interviewees reported that EVIDENT’s activities were not robust enough to warrant such an extensive structure and, furthermore, this structure was never fully operationalised (e.g. the quality assurance board was scarcely utilised).

The EVIDENT coordinating body provided leadership and administrative management across its partners. This body was based at the Institute of Tropical Medicine, which was perceived as logical by interviewees since project funds resided within this partner institute. The coordinator, specifically, was considered a key driving force of EVIDENT and, therefore, critical to the success of EVIDENT. Most participants felt that EVIDENT was well-managed by the coordinating body in terms of handling administrative work, promoting EVIDENT, seeking funding, communicating with partners, keeping every partner engaged, and ensuring equitable participation. However, a few participants stated that the heavy burden of workload on the coordinator, due to lack of personnel within the coordinating body, led to delayed or unclear communication within EVIDENT. They also acknowledged the challenges of leading a collaborative partnership like EVIDENT using words such as “*push and pull*”, “*frustrations*” and “*difficult*”.

Country teams were set up in four African partner institutions to implement case studies and evidence synthesis activities (see Problem-Oriented and Evidence-Informed Decision-Making (POEIDM) – case studies further below). Participants from these teams felt that the flexibility to organise the case studies at country level evoked individual leadership, which evolved and enhanced their personal and work-related functional capacities, particularly by being responsible for structuring and managing a team and in getting tasks completed within the team. This was reflected in the online survey, where almost all respondents were satisfied with country team coordination and most thought this was of high quality. Participants also recognised that they could have done more with this opportunity to take lead, but were limited by the short time frame, lack of expertise, language barriers and shortage of suitable staff.

#### Problem-Oriented Evidence Informed Decision-Making (POEIDM): case studies

Over the course of the project, EVIDENT developed a conceptual framework based on the need to guide case studies in the systematic process of EIDM (Fig. 1). This framework was perceived as logical, simple and easy to understand, and was thought of as an important guide for the project. A series of process notes (*n* = 5) were developed in line with the steps of this framework to further help execute case studies. Interviewees perceived these as good and useful tools, yet also expressed that these were not sufficiently shared amongst the teams.

The four case studies were carried out in Benin, South Africa, Ghana and Ethiopia. Each case study obtained approval from their relevant ethics committees. Based on the conceptual framework, each of these country teams were able to assess the existing pathways of EIDM in their own ways. Fifteen country level nutrition problems were identified from case studies and also during the short courses. Four were prioritised and addressed (one question per case study), using systematic reviews. An additional question was addressed by EVIDENT’s Moroccan partner. Four out of five systematic reviews were in progress with guidance and validation from EVIDENT’s coaches. Meanwhile, Ethiopia produced policy briefs on the effect of palm oil consumption on health and on the exposure of aflatoxin in Ethiopia and outlined policy options to be taken. The case studies were perceived as a useful tool to understand the local policy-making contexts, information needs and decision-making influences.

Interviewees believed engaging stakeholders (decision-makers at country level) was the most critical step of the conceptual framework. It was key to identifying the right questions at the beginning of the stepwise process and ensuring synthesised evidence is used in decision-making, while providing continuous feedback to them on the on-going process. Yet, engaging stakeholders was also the most difficult and time-consuming step. Perceived difficulties included competing interests across local government agencies, the influence of donors on policy-maker agendas, the lack of strong government structures, the absence of existing EIDM systems and the challenge of getting into the circle of decision-makers. Moreover, stakeholders were not eager to participate in this new way of engaging with researchers, had little time, were not willing to share information or a combination of these. This difficulty was further reflected in the limited number of stakeholders engaging with EVIDENT in the completion of the online survey. Nevertheless, the presence of an existing internal network and a good reputation of the partner were mentioned by interviewees to ease the process of stakeholder engagement.

As they carried out the steps of the EIDM process (Fig. 1), country teams acknowledged the importance of understanding all influencing factors in EIDM, including the importance of contextualisation, and the need for local economic evaluations. These steps were perceived by them as ‘difficult’ and time-consuming, and not always feasible as the project was an add-on to partners’ daily (high) workload. In addition, the capacity to carry out the stepwise process and to do a systematic review had to be built along the way. A number of other barriers were also identified, including ambiguity in deliverables (see Governance and Leadership further below), high expectations versus available funding, insufficient commitment from partners, contending with stakeholders’ different ways of working, bureaucracy, and operational issues such as electric power cuts, sub-optimal internet connectivity and lack of access to scientific databases. These barriers contributed to the delay in producing evidence synthesis products, wherein, at the time of evaluation, the steps of contextualisation and recommendations had not yet been reached. Nonetheless, various other outputs were achieved, including presentations at various conferences, a publication in the Regional Strategic Analysis and Knowledge Support System Annual Trends and Outlook Report 2015, a blog on the EVIDENT partnership, articles in the Scaling Up Nutrition (SUN) Movement’s In Practice Brief, and three other systematic reviews aside from those planned within the case studies (*n* = 4) and that by EVIDENT’s Moroccan partner (*n* = 1) [8, 18, 28–30].

#### Horizontal collaboration

##### Network and communication

EVIDENT was perceived by interviewees as an active and functional network of partners (group I). Almost all survey respondents felt satisfied with their experience of working within the collaborative partnership. They felt at ease to discuss any issues and felt that they were listened to. This was in part attributed to partners’ existing relationships with each other prior to the project, and their shared values.

EVIDENT provided networking opportunities, allowing partners to work together on EIDM as an organised group rather than only as an individual, to share knowledge, build friendships, connect with each other, and to support and help each other across countries. It helped build partnerships outside of EVIDENT and establish new kinds of partnerships such as those with other academics and agencies (e.g. the International Food Policy Research Institute (IFPRI)), within African nutrition institutions, between researchers and policy-makers, and outside of the nutrition field. Some interviewees believed that having this international network also gave African participants the opportunity to become more visible and to build academic reputation.

However, an imbalance in network interactions was perceived by some interviewees, wherein a high proportion of the communication and interaction towards country teams and potential funders and agencies was found to originate from the coordinating body (see Governance and Leadership further below) and other European participants. Some interviewees reported that they were often unaware of the communication between the coordinating body and potential funders. Moreover, country teams felt they had less influence with donors and agencies, who are typically based in the Global North and thus they could not meet them easily.

Multiple media were used for communication within EVIDENT, including face-to-face meetings, email, Skype, Cisco WebEx, text messaging (via WhatsApp), telephone calls and the project website. Face-to-face communication was most preferred by survey respondents. The intensity of communication, participant engagement and momentum of action was greatest during face-to-face meetings but usually declined in-between these meetings. Due to the time and financial constraints of meeting in person, web-based communication, particularly email, was mostly used. One interviewee highlighted the use of email as a limitation because this medium assumes that people will read and respond to them. Skype calls were limited due to sub-optimal internet connectivity in African settings (see POEIDM – case studies further below), and survey respondents felt that the EVIDENT website was less useful for communication as it was not updated frequently enough. Some participants felt that the physical distance, geographical isolation, and unclear goals and deliverables, made it difficult to communicate, particularly when working via email and with partners they did not know very well.

Some coaches perceived a lack of initiative from the country teams towards them, while some country teams felt there was a lack of understanding between the coaches and the country teams. This was thought to contribute to the slow and/or insufficient responses from coaches. Limited interaction was also observed across country teams as participants reported that they did not know what other case studies were working on. Reasons for limited interactions were identified during interviews and included the time needed to build trust, where it is more difficult for African partners to work together and have faith in one another than it is for them to work with partners in the North, and not working simultaneously on the same stages as other case studies.

##### Visibility

A project website and participation in 12 international conferences, the latter of which was originally unplanned, showcased EVIDENT’s activities and increased its visibility. Almost half of the interviewees perceived these efforts to have championed the mission of EVIDENT to an international audience. A total of 75% of survey respondents felt these visibility efforts worked “*well*” or “*fairly well*”. The coordinating body played a critical role in talking to external stakeholders such as potential funders, including the SUN Movement, and IFPRI. Yet, visibility efforts were perceived as limited due to insufficient funding allocated to visibility activities.

##### Sustainability

EVIDENT ensured project sustainability by building the capacity of local researchers in Africa in EIDM, and by securing additional funds through Agriculture for Nutrition and Health Research at IFPRI to write a protocol for large-scale funding. These funds were to be used by EVIDENT’s Ghanaian and South African partners, who felt ready to step up as coordinators from 2017 onwards and continue EVIDENT’s work*.*

Despite these successes, interviewees and survey respondents both felt that EVIDENT had not become sufficiently sustainable. Almost all participants perceived additional funding as a critical factor to ensuring the sustainability of EVIDENT as, without funding, EVIDENT could not continue. Interviewees felt that, to obtain funds, there should have been outputs based on intended objectives such as evidence synthesis products. However, EVIDENT experienced delays in producing these planned outputs (see POEIDM – case studies further below).

### Improving

#### Capacity-building

To tackle the time challenge of incorporating evidence synthesis activities into daily work, interviewees proposed promoting local courses by incorporating the modules into undergraduate and Master’s programs in nutrition of partner countries and using more e-learning facilities, whereas increasing staff exchange between partners was also suggested to enhance learning opportunities. Interviewees also recommended the need for training in other practical aspects of EIDM such as leadership, lobbying and engaging with decision-makers, and in building functional capacity such as how to search databases, develop country teams or use guidance tools (e.g. process notes).

#### Governance and leadership

Participants called for a simpler management structure with a clearer sense of direction, investment in better communication, training in leadership capacities, and more support and mentorship to improve governance and leadership within EVIDENT overall. Hiring sufficient staff to share the heavy workload and a rotation system, where after every few years, a different institution takes on the responsibility of coordination and management, was also recommended.

#### POEIDM – case studies

The conceptual framework could be improved by taking real life situations into account, and adapting it, together with the process notes, to the local contexts and by incorporating lessons from similar initiatives. To minimise the duration for delivering on decision-maker information requests, performing a rapid review process, or generating shorter term evidence synthesis products was suggested by interviewees as alternatives to systematic reviews.

#### Horizontal collaboration

##### Network and communication

To improve as a collaborative partnership, EVIDENT requires better communication. For this, regular updates, face-to-face contact and the use of diverse communication streams, such as newsletters, conferences and articles, were recommended. It was further advised that coaches should be given a dedicated teaching role in the short courses to facilitate, and increase, interactions between coaches and trainees. These enhanced interactions will build better cross-continent teams, thereby bridging barriers between partners, and enhance learning opportunities from shared experiences. Sufficient buy-in from all participants would also encourage interactions within the network. Linking, and collaborating, with more existing organisations/entities, and thereby having access to more human resources and more stakeholders, would improve functionality. Meanwhile, a need for results and finished evidence synthesis products was also identified to improve communication and better disseminate information to external stakeholders. To be able to achieve and coordinate these suggestions, hiring dedicated staff to manage communication for EVIDENT was considered an important part of the next steps forward.

##### Visibility

Recommendations to improve EVIDENT’s visibility efforts include linking and strengthening partnerships with EVIDENT stakeholder organisations such as SUN Movement, IFPRI and PRICELESS, while creating opportunities for additional countries to become involved in the project. At country level, and particularly with decision-makers, visibility can be enhanced through local seminars, workshops and meetings. Sufficient funding is needed for these recommendations, particularly to support mobility and to hire staff to manage visibility activities on behalf of EVIDENT.

##### Sustainability

Continuation of capacity-building was perceived as essential to allow African researchers to be less dependent on EVIDENT’s northern expertise and/or other initiatives for local EIDM processes. A need to contextualise activities from the beginning, and to foster and create ownership was identified. Working together more, as a collective, on project activities would help generate this ownership. Several interviewees felt that there was a need to set out new goals and objectives for the continuation of EVIDENT.

## Discussion

Over the past 3 years, EVIDENT successfully established a functional and collaborative partnership across the Global North and South. EVIDENT built the capacity of African researchers in EIDM in order to identify, and address, the information needs of local decision-makers in nutrition and health. This capacity, along with a conceptual framework and process notes, facilitated the understanding of EIDM processes in four different African contexts. In addition, EVIDENT enhanced its visibility on an international level, and successfully secured its continuation by transferring project leadership to its Ghanaian and South African partners for future activities. Lessons learned from these activities can be used to inform improvements in EVIDENT, and in EIDM in nutrition overall.

EVIDENT’s flexibility, in being able to adapt to its partners’ requirements, was a key strength of the project. When partners identified a critical gap in evidence synthesis and knowledge management capacities, which affected their ability to respond to project objectives, EVIDENT responded by providing tailored short courses and building capacity along the way; this, in turn, ensured buy-in from the partners. In addition to this capacity, participants also highlighted the need for basic technical skills such as how to search online libraries, use guidance tools and form country teams. The identification of the lack of capacity at different levels is not new, and has been previously highlighted as a limitation to EIDM [31]. On the other hand, project flexibility also contributed to ambiguity in EVIDENT’s objectives and deliverables. Therefore, having additional staff dedicated to managing and coordinating EVIDENT’s activities, by way of sufficient funds, would eliminate this uncertainty. There may be different options to achieve improved communication depending on the context. One option is to reduce the task burden of current staff by hiring additional staff if this makes sense in a particular context. Elsewhere, the best option may require enhancement of communication competencies of existing staff. However, the decision should be informed by the context and the need to ensure sustainability whilst maximising benefit from existing resources.

Various alternative suggestions for addressing the capacity gap were identified in this evaluation. In the short-term, EVIDENT modules can be translated into e-learning courses, which are an economical alternative to attending face-to-face short courses [32, 33]. These courses are typically self-paced, use diverse streams of interactive multimedia, and allow a larger and wider cohort of stakeholders’ access to an international platform for learning and sharing experiences [33–35]. Many such e-learning courses are already available (e.g. the eNutrition Academy, ‘Health Technology Assessments: Choosing which Treatments get Funded’ and ‘Measuring and Valuing Heath’ by The University of Sheffield) [36–38]. Strategic partnerships with such knowledge initiatives can be beneficial in achieving the capacity-building and sustainability recommendations of EVIDENT and EIDM across LMICs; however, for this option, sufficient and stable internet connectivity has been identified as a limitation [33–35].

E-learning courses should be offered alongside the longer-term suggestion of integrating modules into formal education programmes at university level in LMICs [39]. These modules can be incorporated in nutrition and nutrition-related courses (e.g. food science, agriculture, ethnography) and be supplemented with technical capacities in financing, statistics, programme planning and implementation, as well as functional capacities in writing, team building, leadership, lobbying, advocacy and negotiation, behavioural science, communication, and ethics [39]. Such integration provides additional opportunities to update, and renew, existing Higher Education Institution programme curricula [24] and to ensure availability of all-round capacity in addressing the influencing factors of decision-making processes.

Despite some challenges with communication and time, the genuine partnership between the Global North and South, in jointly delivering short courses and providing coaching, proved to be highly valued. Bringing researchers together from LMICs and HICs helped share and maximise expertise, experiences and perspectives. This blended learning technique proved effective, and the success of this approach confirms what has been previously suggested in the literature [39–42].

EVIDENT recognised the need for leadership skills in engaging stakeholders resolving conflict, and working better as a team in multi-cultural and multi-sectoral contexts [9, 43]. Furthermore, evidence use in decision-making is enabled by strong leadership and ‘soft’ persuasion skills [44, 45]. Local leadership capacities can be developed via programmes that are grounded in practical application and learn-by-doing approaches (e.g. the African Nutrition Leadership Programme) [39, 46]. This is especially important, as much of the current leadership in research typically comes from external academic institutions in HICs [42].

Stakeholder engagement was identified by country teams as difficult and yet the most important aspect of EIDM. Shroff et al. [9] resonated with this difficulty – getting researchers and policy-makers that have never worked together to team up and hold dialogue is a difficult and slow process. It was, however, crucial to involve stakeholders from the beginning, and throughout the entire stepwise process, to align priorities and foster a common vision towards decision-making [45, 47]. This would therefore facilitate the uptake of synthesised evidence [45, 48]. Early stakeholder involvement means that evidence synthesis products are more easily perceived as clear and unambiguous by policy-makers [9, 45]. Informal relationships amongst stakeholders can play an important role in easing the process of EIDM [4, 5, 9]. These pre-existing, and trusting, relationships between researchers and policy-makers can translate into trust in the synthesised evidence, and therefore boost their use in policy-making [49].

EVIDENT succeeded in part in providing its partners and stakeholders with opportunities to collaborate, share knowledge and strengthen relationships. Similarly, it also provided support and guidance in establishing new partnerships with other agencies (e.g. IFPRI, Africa Evidence Network, SUN Movement) and African nutrition institutions, and in bridging the gap between researchers and decision-makers. These global collaborations and horizontal links increased partners’ visibility and credibility. A key gap, however, was the absence of a heterogeneous group of people with multi-sectoral backgrounds; 73% of directly involved participants worked in nutrition, and almost all were operating under research and academia. While disparity between the sectors is the norm in Africa, an overlap in backgrounds is also common as there are too few skilled people working in nutrition; the distinction in stakeholder roles are not as clear-cut as they are in HICs [9]. For these reasons, EVIDENT could have experienced difficulties in engaging and collaborating with stakeholders. EVIDENT requires more heterogeneity across its partners and stakeholders. More opportunities for interactive workshops and meetings with diverse stakeholders are also required to facilitate vertical collaborations within countries (e.g. direct interaction with the public via social networking and technology-driven platforms such community participatory videos) [39].

Participants realised that, like stakeholder engagement, the contextualisation of EIDM processes to local settings should also be fostered from the beginning. Shroff et al. [9] show that strategies focusing solely on providing evidence to decision-makers are insufficient to enable EIDM. EVIDENT identified a need to take all influencing factors (e.g. bureaucracy, cultural habits and affordability) and different ways of working into consideration; Innvaer [4], Orton [5], Oxman [10] and Nabyonga-Orem [49] detail a number of these key influences.

Time constraints were frequently reiterated as a barrier to EIDM activities across EVIDENT. The stepwise process, comprising the systematic review, was arduous and time-consuming for country teams; while systematic reviews are rated the highest quality of evidence, different stakeholders consider different types and aspects of evidence important [50, 51]. A full systematic review, for example, can take up to year to complete [50]. Therefore, EVIDENT did not produce many planned evidence synthesis outputs. To tackle these time constraints, quicker alternatives such the rapid reviews should be considered [50–52]. Yet, to fully understand EIDM in a specific context, with the given challenges and while building trusting relationships with stakeholders, requires both time and commitment.

Partners were also involved in efforts to secure additional funding for EVIDENT’s continuation, alongside the process of producing evidence synthesis outputs. These outputs were to be used as proof of concept when approaching donors, and were therefore identified as crucial to the sustainability of EVIDENT. However, both are time-intensive activities and it was therefore difficult to accomplish both at the same time. Longer duration would allow enough time to ensure the sustainability of EVIDENT in the future, since long-term funding is needed to be able to advance more in this area of work. Again, participants stressed the institutionalisation of such a knowledge platform and providing a link with government entities. Previously, such links have been confirmed to be effective [53, 54].

The low participation rate of indirectly involved individuals was a limitation of this evaluation. This rate, however, highlights the difficulties faced by EVIDENT partners in engaging stakeholders. As EVIDENT set up the partnership and is still gauging EIDM activities in the Global South over the 3-year period, a second evaluation of the project’s growing network and activities is recommended in a few years. Meanwhile, almost all directly involved partners took part in this evaluation through the different instruments of data collection.

The reported findings have applicability for settings that are similar to the ones in Africa, where EVIDENT was implemented. It is therefore proposed that future initiatives on EIDM should consider the approach that EVIDENT has employed. A critical condition of its application is that it is important to consider the local setting and its peculiar circumstances in order to appropriately adapt the EVIDENT EIDM frame since the current evaluation demonstrates that EIDM may operate differently depending on the context. The case study approach that was applied in EVIDENT can serve as a useful testing phase at country level to determine the best approach to initiate EIDM. It will also be important to consider the challenging aspects of collaborative work and the lessons learned as a result, as indicated in our earlier publications [7, 8].

## Conclusion

Taken altogether, the findings are consistent with the a priori EIDM framework (Fig. 1) developed by EVIDENT to guide its activities as. In its first 3 years, EVIDENT established a collaborative partnership of researchers, national and international decision-makers, and other stakeholders across the Global North and South. This partnership supported the processes of building the capacity of African researchers in EIDM processes. A part of this process involved engagement with local decision-makers in identifying and addressing locally relevant nutrition and health-related decision-making questions. These questions were addressed through case studies in four different African country settings. Implementation of the case studies has provided insight into the practical aspects of implementing EIDM. Several call-for-action points for future activities within EVIDENT, and in EIDM in nutrition in general, have been identified through this process.

First, innovative approaches, which combine blended learning techniques and technology, and are also sustainable, are required for long-term capacity-building in LMICs. Second, investment in stakeholder engagement is critical to ensuring the use of EIDM practices. Stakeholders must be engaged throughout the stepwise process, at all levels (vertical) and across all sectors (horizontal). Building a global, and collaborative, network of heterogeneous stakeholders is useful for guidance and support in EIDM. This can be attained by establishing new connections but also by strengthening existing relationships. Finally, utilisation of the practical experiences of the case studies in implementing the stepwise process to develop context-specific guidance can facilitate the uptake of EIDM processes.

Box 1 EVIDENT’s objectives and activities The EVIDENT partnership comprised European and African partners with long-standing institutional collaborations. Activities kicked off in January 2014 with the following objectives:

- Enhance capacity and leadership of African researchers and decision-makers in knowledge management and translation by providing high-quality methodological training and support;
- Create in-country collaboration between decision-makers and scientists to improve their ability to both articulate their research needs and appropriately use evidence;
- Address nutrition and health-related questions posed by African stakeholders in a timely and transparent manner;
- Create a global collaboration of scientists and decision-makers committed to working together and share experiences in the application of the principles and processes of EIDM in nutrition;
- Foster global collaborations to share existing knowledge and generate new knowledge and competencies, where necessary, to inform national and regional nutrition policy.

To achieve these objectives, the partners developed a conceptual framework around the systematic process for EIDM in nutrition (Fig. 1). The pathway, developed in response to expressed needs, comprised a variety of complex processes, including identifying priority policy/programme-related issues as expressed by decision-makers, performing evidence synthesis, adapting the best available evidence to the local context and country-specific needs, and creating an enabling environment to drive the policy process forward based on the contextualised recommendations. The close involvement of decision-makers throughout the entire process was anticipated as an important and unique aspect.

Anchored at country level, there were three main pillars along the pathway, namely (1) Problem-oriented and EIDM (POEIDM), (2) capacity strengthening and leadership and (3) horizontal collaboration within and across partner countries. Figure 1 presents the overall conceptual framework of EVIDENT.

Four of the partner countries – Benin, Ethiopia, Ghana and South Africa – implemented case studies based on the conceptual framework. Through activities set under each pillar, EVIDENT investigated whether such a stepwise process for identifying and using evidence would lead to better decision-making and better nutrition policies in countries with a high burden of malnutrition. Case studies allowed EVIDENT to explore the best conceptual representation of how these processes would work across countries and learn whether the a priori framework applies in a linear way, as proposed in Fig. 1, or whether it is a more iterative process. The studies also helped identify knowledge, capacity and leadership gaps and to inform developments in EIDM in nutrition in Africa.

From 2014 to 2016, EVIDENT was funded by the Belgian Development Cooperation and Nutrition Third World.

Box 2 Case study of evidence-informed decision-making implementation in Ghana [8] **Capacity development**

Two Ghanaian researchers from the University of Ghana participated in the first EVIDENT short course on systematic reviews in 2014. Subsequently, in 2015, the two researchers also participated in the second EVIDENT short course on ‘Translating Evidence in Nutrition into Country-Specific Recommendations’.

**Evidence synthesis**

As part of the systematic reviews short course, researchers identified a topic of relevance to Ghana to conduct a systematic review on. The systematic review question was to, ‘identify the linkage between exclusive breastfeeding duration and adequacy of complementary feeding among infants in low- and middle-income countries’. This question was motivated by documented maternal perception that exclusive breastfeeding for 6 months adversely affects transition to complementary feeding. The systematic review protocol was registered and subsequently carried out.

**Stakeholder mapping and prioritisation**

Beginning in 2015, the researchers implemented a case study focused on answering two questions, namely ‘how are nutrition priorities determined in Ghana?’ and ‘what are the opportunities and gaps for utilising evidence for decision-making in nutrition in Ghana?’ Data for the case study was obtained through a combination of desk review of existing literature, stakeholder mapping (using social net-mapping technique), and in-depth interviews with key informants across various agencies in government, civil society, academia, donors and united nations agencies working to address nutrition in Ghana. The findings as well as the lessons learned from the case study were shared with key stakeholders in Ghana.

## Additional files

Additional file 1: COREQ Checklist. A completed COREQ checklist. (PDF 423 kb)

Additional file 2: Semi-structured questioning route for group I. A questioning route used to guide the in-depth interviews of group I participants. (DOCX 36 kb)

Additional file 3: Semi-structured questioning route for group II. A questioning route used to guide the in-depth interviews of group II participants. (DOCX 35 kb)

Additional file 4: Online survey questions for groups I and II. Questions online survey that sent to directly involved participants (groups I and II). (PDF 608 kb)

Additional file 5: Online survey questions for group III. Questions from the shorter online survey that was sent to indirectly involved participants (group III) (PDF 105 kb)

Additional file 6: Node tree for the analysis of in-depth interviews. A node tree of code, analyse and summarise in-depth interviews (DOCX 899 kb)

Additional file 7: Matrix of supporting data. A matrix illustrating data triangulation from EVIDENT documentation, online surveys, in-depth interviews and the participatory discussion (DOCX 70 kb)

## Abbreviations

EIDM: evidence-informed decision-making
EVIDENT: Evidence-informed Decision-making in Nutrition and Health
HICs: high-income countries
IFPRI: International Food Policy Research Institute
LMICs: low- and middle-income countries
POEIDM: problem-oriented and evidence-informed decision-making
SUN: Scaling Up Nutrition

## References

[1] Ioannidis JPA, Greenland S, Hlatky MA, Khoury MJ, Macleod MR, Moher D (2014). Increasing value and reducing waste in research design, conduct, and analysis. Lancet.

[2] Dobrow M, Goel G, Upshur R (2004). Evidence-based health policy: context and utilisation. Soc Sci Med.

[3] Development Initiatives (2017). Global Nutrition Report 2017: Nourishing the SDGs.

[4] Innvaer S, Vist G, Trommald M, Oxman A (2002). Health policy makers’ perceptions of their use of evidence: a systematic review. J Health Serv Res Policy.

[5] Orton L, Lloyd-Williams F, Taylor-Robinson D, O’Flaeherty M, Capewell S (2011). The use of research evidence in public health decision making processes: systematic review. PLoS One.

[6] Bowen S, Erickson T, Martens PJ, Crockett S (2009). More than “using research”: the real challenges in promoting evidence-informed decision-making. Healthc Policy.

[7] Aryeetey R, Holdsworth M, Taljaard C, Hounkpatin WA, Colecraft E, Lachat C (2017). Evidence-informed decision making for nutrition: African experiences and way forward. Proc Nutr Soc.

[8] Holdsworth M, Aryeetey R, Jerling J, Taljaard C, Nago E, Colecraft C, Covic N, Hendricks SL (2016). The challenges, opportunities, and lessons learned in evidence-informed decision making in Africa. Achieving a nutrition revolution for Africa: The road to healthier diets and optimal nutrition.

[9] Shroff Z, Aulakh B, Gilson L, Agyepong IA, El-Jardali F, Ghaffar A (2015). Incorporating research evidence into decision-making processes: researcher and decision-maker perceptions from five low- and middle-income countries. Health Res Policy Syst.

[10] Oxman A, Lavis J, Lewin S, Fretheim A (2009). SUPPORT tools for evidence-informed health policy making (STP 1): what is evidence-informed policymaking?. Health Res Policy Syst.

[11] Resnick D, Babu S, Haggblade S, Hendriks S, Mather D (2015). Conceptualizing Drivers of Policy Change in Agriculture, Nutrition, and Food Security: The Kaleidoscope Model. IFPRI Discussion Paper 1414.

[12] Gillespie S, Menon P, Kennedy AL (2015). Scaling up impact on nutrition: what will it take?. Adv Nutr.

[13] CGIAR: Research Program on Agriculture for Nutrition and Health. 2017. http://a4nh.cgiar.org/. Accessed 1 Jan 2018.

[14] LANSA Leveraging Agriculture for Nutrition in South Asia. 2013. http://lansasouthasia.org/. Accessed 1 Jan 2018.

[15] Beyero M, Hodge J, Lewis A (2015). Leveraging Agriculture for Nutrition in East Africa (LANEA) Country Report – Ethiopia.

[16] INASP. Building Capacity to Use Research Evidence (VakaYiko). 2019. https://www.inasp.info/project/building-capacity-use-research-evidence-vakayiko. Accessed 19 Jan 2019.

[17] Covic N, Hendriks SL (2016). Achieving a Nutrition Revolution for Africa: The Road to Healthier Diets and Optimal Nutrition. ReSAKSS Annual Trends and Outlook Report 2015.

[18] Scaling Up Nutrition Movement. From Science to Action: Academia and Decision-Makers Unite in SUN Countries. Scaling Up Nutrition In Practice Brief. 2016. https://scalingupnutrition.org/wp-content/uploads/2016/06/InPractice_Science_no05_ENG_20160609_web_pages.pdf. Accessed 19 Jan 2019.

[19] Africa Evidence Network. 2014. http://www.africaevidencenetwork.org/about-us/. Accessed 1 Jan 2018.

[20] Morris SS, Cogill B, Uauy R, Maternal and Child Undernutrition Study Group (2008). Effective international action against undernutrition: why has it proven so difficult and what can be done to accelerate progress?. Lancet.

[21] EVIDENT: Evidence-informed decision-making in health and nutrition. 2017. http://www.evident-network.org/. Accessed 1 Jan 2018.

[22] Lachat C, Nago E, Roberfroid D, Holdsworth M, Smit K, Kinabo J (2014). Developing a sustainable nutrition research agenda in sub-Saharan Africa—findings from the SUNRAY Project. PLoS Med.

[23] Tong A, Sainsbury P, Craig J (2007). Consolidated criteria for reporting qualitative research (COREQ): a 32-item checklist for interviews and focus groups. Int J Qual Assur Health Care.

[24] Gillespie S, Haddad L, Mannar V, Menon P, Nisbett N, Maternal and Child Nutrition Study Group (2013). The politics of reducing malnutrition: building commitment and accelerating progress. Lancet.

[25] Krueger R, Casey MA (2000). Focus Groups: A Practical Guide for Applied Research.

[26] Lester S (1999). An Introduction to Phenomenological Research.

[27] Guijt I (2014). Participatory Approaches, Methodological Briefs: Impact Evaluation 5.

[28] Public Health Topics. Focus on Nutrition in Africa. 2017. https://publichealthtopics.wordpress.com/page/4/. Accessed 1 Jan 2018.

[29] Gissing SC, Pradeilles R, Osei-Kwasi HA, Cohen E, Holdsworth M (2017). Drivers of dietary behaviors in women living in urban Africa-a systematic mapping review. Public Health Nutr.

[30] Verstraeten R, Hawwash D, Lachat C, Bonn N, Pinxten W, Gillespie S, Holdsworth, Booth A. Nutrition prioritisation: three inter-dependent (mapping, methodology, and ethics) systematic review outputs. 2016. PROSPERO Registration: CRD42016043805. https://www.crd.york.ac.uk/PROSPERO/display_record.php?RecordID=43805. Accessed 1 Jan 2019.

[31] Yamey G. What are the barriers to scaling up health interventions in low and middle income countries? A qualitative study of academic leaders in implementation science. Glob Health. 2012;8:11. 10.1186/1744-8603-8-11. 10.1186/1744-8603-8-11 PMC3514334 22643120

[32] Downes S. Connectivism and Connective Knowledge: Essays on Meaning and Learning Networks. National Research Council Canada. 2012. https://www.downes.ca/files/books/Connective_Knowledge-19May2012.pdf. Accessed 21 Jan 2019.

[33] Vacanti F, Ciaperoni S, Perifanou M, Bekiaridis G, Martinez I, Gomez P, Talmo TM, Mitchell M, Inayat M. Language Massive Open Online Courses Research report on MOOCs Pedagogical Framework. 2015. https://cesie.org/media/LangMOOCs-research-report.pdf. Accessed 1 Jan 2019.

[34] Liyanagunawardena TR, Williams SA (2014). Massive open online courses on health and medicine: a review. J Med Internet Res.

[35] Goldberg LR, Crocombe LA (2017). Advances in medical education and practice: role of massive open online courses. Adv Med Educ Pract.

[36] Nutrition Academy. Assessment of Dietary Intake for Individuals. 2014. http://www.enutritionacademy.org/courses/assessment-of-dietary-intake-for-individuals/. Accessed 1 Jan 2018.

[37] FutureLearn. Health Technology Assessment: Choosing Which Treatments Get Funded. 2017. https://www.futurelearn.com/courses/hta#section-overview. Accessed 1 Jan 2018.

[38] FutureLearn: Measuring and Valuing Health. 2017. https://www.futurelearn.com/courses/valuing-health#section-overview. Accessed 1 Jan 2018.

[39] Fanzo JC, Graziose MM, Kraemer K, Gillespie S, Johnston JL, de Pee S (2015). Educating and training a workforce for nutrition in a post-2015 world. Adv Nutr.

[40] Clark D (2003). Blended Learning: An EPIC White Paper.

[41] Shrimpton R, Hughes R, Recine E, Mason JB, Sanders D, Marks GC (2014). Nutrition capacity development: a practice framework. Public Health Nutr.

[42] Ndounga Diakou LA, Ntoumi F, Ravaud P, Boutron I (2017). Published randomized trials performed in Sub-Saharan Africa focus on high-burden diseases but are frequently funded and led by high-income countries. J Clin Epidemiol.

[43] Harries U, Elliot H, Higgins A (1999). Evidence-based policy-making in the NHS: Exploring the interface between research and the commissioning process. J Public Health Med.

[44] Teng F, Mitton C, MacKenzie J (2007). Priority setting in the provincial health services authority: survey of key decision makers. BMC Health Serv Res.

[45] Turner S, D’Lima D, Hudson E, Morris S, Sheringham J, Swart N (2017). Evidence use in decision-making on introducing innovations: a systematic scoping review with stakeholder feedback. Implement Sci.

[46] African Nutrition Leadership Program. 2017. https://www.africanutritionleadership.org/. Accessed 1 Jan 2018.

[47] Rycroft-Malone J, Seers K, Chandler J, Hawkes CA, Crichton N, Allen C (2013). The role of evidence, context, and facilitation in an implementation trial: implications for the development of the PARIHS framework. Implement Sci.

[48] Ahmad R, Kyratsis Y, Holmes A (2012). When the user is not the chooser: learning from stakeholder involvement in technology adoption decisions in infection control. J Hosp Infect.

[49] Nabyonga-Orem J, Mijumbi R (2015). Evidence for informing health policy development in Low-income Countries (LICs): perspectives of policy actors in Uganda. Int J Health Policy Manag.

[50] Schünemann HJ, Moja L (2015). Reviews: Rapid! Rapid! Rapid! … and systematic. Syst Rev.

[51] Booth A, Sutton A, Papaioannou D (2016). Systematic Approaches to a Successful Literature Review.

[52] Ganan R, Ciliska D, Thomas H (2010). Expediting systematic reviews: methods and implications of rapid reviews. Implement Sci.

[53] Alliance for Health Policy and Systems Research (2012). Fellowship Programme on Monitoring and Evaluation Methodology for Evidence-to-policy Initiatives.

[54] Alliance for Health Policy and Systems Research (2013). Evidence-informed Policy Making - An Analysis of Approaches Under the Sponsoring National Processes and Enhancing Capacity to Apply Research Programs.

