# Supplementary material for: Lessons learned from Evidence-Informed Decision-Making in Nutrition & Health (EVIDENT) in Africa: a project evaluation
Source: Health Res Policy Syst. 2019 Jan 31;17:12. doi: 10.1186/s12961-019-0413-6 (PMC6357392; doi:10.1186/s12961-019-0413-6)
Supplement: Supplementary file 2 — Semi-structured questioning route for group I. A questioning route used to guide the in-depth interviews of group I participants. (DOCX 36 kb) [file 12961_2019_413_MOESM2_ESM.docx]

**Additional file 2** Semi-structured questioning route for group I

| **Components** | | | **Relevant Evaluation Questions** |
| --- | --- | --- | --- |
| **Description of participant** | | | |
| **Involvement in EVIDENT** | 1. Could you tell me about how you became involved with EVIDENT?   *Probe: How did you become a partner?*   1. What was your motivation to become and stay involved in EVIDENT?   *Probe: for your own benefit, for the benefit of the EVIDENT process?* | | |
| **Strengthening capacity & leadership** | | | |
| **Capacity building** | EVIDENT carried out a number of trainings to strengthen its partners’ capacity in the process of EIDM.  Did you participate in these trainings? Or were you involved in another way? (in order to choose 3A or 3B)   1. A) For interviewees who participated in the training:   What was the value of the trainings for you, in performing the work that was required within EVIDENT?  *Probe: personal/professional; skillset obtained?*  B) For interviewees who assisted the training (organise, coach):  What was the value of the trainings for the work that was required within EVIDENT?   1. Beyond this training, what other types of capacity building exercises would have been useful within EVIDENT?   ***!! Probe****: How could capacity building be improved within EVIDENT?* | | |
| **Leadership** | EVIDENT demanded leadership from its partners in order to achieve its objectives.   1. How has EVIDENT enhanced your own leadership skills?   *Probe: What was needed from you to lead the project forward in influencing EIDM in your own setting?*   1. What leadership emerged amongst partners or other actors?   ***!! Probe****: describe these: horizontal/vertical, North-South/South-South/North-North, or within countries*   1. How could leadership capacities have been enhanced more amongst partners? | | |
| **Problem-oriented and Evidence-informed Decision-making** | | | |
| **Steps in the conceptual framework:**   1. **stakeholder involvement** 2. **prioritisation of research questions** 3. **evidence products generation** 4. **evidence products contextualisation** 5. **facilitation of evidence** | | **Conceptual framework:**  EVIDENT has developed a conceptual framework to achieve its objectives, from mapping stakeholders to the facilitation of evidence.   1. What are your views on this conceptual framework for EIDM?   ***!! Probe****: What have been your experiences (positive and negative) regarding each step in the framework (stakeholder involvement, prioritisation of research questions, evidence products generation, evidence products contextualisation, facilitation of evidence)?*   1. Could you suggest improvements for the conceptual framework? | |
|  |  | **Guidelines:**  As one of its objectives, EVIDENT intended to develop a series of guidelines to facilitate stakeholder mapping, prioritisation of a research question, evidence synthesis products ( such as systematic review, policy brief, and publication guidelines)   1. What are your views on these guidelines in terms of usefulness, issues, challenges and opportunities? | |
| **Case country studies** | | Are you involved in a case country study?  No > If South partner > A; If North partner> Go to Q17;  Yes > In which way? If member > B; if coach (or other) > C  **A) If South partner and NOT a member of a case country study:**   1. What were the reasons for you not to commit to the implementation of the case studies? 2. **If a member of a case country study:** 3. As a person involved in a case country study, what has been your experience in setting up the country study?   ***!! Probe****: What factors have helped facilitate the process and what barriers/implementation challenges have you faced?*   1. How has mapping stakeholders helped in understanding the nutrition landscape in your country? 2. Can you describe your experience with identifying and engaging with stakeholders within your country? 3. How could the process of generating outputs such as systematic reviews and policy briefs of the conceptual framework be improved and streamlined? 4. **If coach/facilitator of a case country study:** 5. What has been your experience in facilitating the SR of the country study? 6. How could the process of generating outputs such as systematic reviews and policy briefs of the conceptual framework be improved and streamlined? | |
| **Horizontal Collaboration/Network** | | | |
| **Communication**  **& Visibility** | | EVIDENT developed a communication strategy to streamline communication within the collaboration and to increase its visibility.   1. What are your views on EVIDENT’s communication strategy?   *Probe: any challenges faced when communicating and engaging with others?* | |
|  |  | 1. What are your views on EVIDENT’s visibility? 2. How could communication and visibility both be improved? | |
| **Network** | | 1. What has been the added value of EVIDENT operating as a network of partners from North-South, South-south, in-country? 2. How functional was the EVIDENT network in your setting? 3. Could you suggest a better format for EVIDENT, other than a network of partners?   *Probe: e.g. institution, …* | |
| **MANAGEMENT** | | | |
| **Communication** | | 1. How did you perceive the management’s communication towards partners and also towards external stakeholders such as funding agencies, the SUN movement, IFPRI and others? | |
| **Operationalisation** | | EVIDENT has an outlined management structure including different bodies like the expert panel (coaches), the coordination body, the country teams, etc.   1. What are your views on the advantages and disadvantages of this overall structure? 2. What are your views on the functioning of EVIDENT’s coordination body?   *Probe: pros and cons of the management structure?* | |
| **Financial** | | 1. What are your views on the funding of EVIDENT?   *Probe: How did EVIDENT look for financial support?* | |
| **SUSTAINABILITY** | | | |
| Many initiatives like EVIDENT have failed to reach sustainability (e.g. SURE). EVIDENT had as an objective to become a sustainable project at the end of the current seed funding.   1. What are your views on EVIDENT’s way to ensure its sustainability?   *Probe: what efforts did EVIDENT do to ensure sustainability?*   1. What should be done to increase EVIDENT’s sustainability?   *Probe: what are the elements needed to make EVIDENT a leading institution in the EIDM process?*   1. Why do you think EVIDENT should continue to exist? | | | |
| **LESSONS LEARNED (barriers, drivers, opportunities, enabling environment)** | | | |
| EVIDENT is ongoing for almost 3 years now. EVIDENT would have experienced success but also faced a number of challenges over the course of three years.   1. What are the lessons you have learned from working with EVIDENT? 2. What are EVIDENT’s biggest weaknesses?   *Probe: what were the issues you faced?*   1. What are EVIDENT’s biggest strengths? | | | |
| **UNINTENDED CONSEQUENCES** | | | |
| EVIDENT had set out its goals from the beginning, but developed in a very dynamic manner, take for example the kick-off meeting in which the management structure was decided by all partners; so next to the foreseen activities also other things could have taken place.   1. What unintended initiatives or events took place over the past 3 years of EVIDENT?   *Probe: could you elaborate a bit further on…?* | | | |

We would like to reach a snowballing effect in this evaluation in which participants name other people who might be related to EVIDENT in their setting. So could you please name 2 or 3 other stakeholders that have been associated with EVIDENT in your setting? Would you be willing to provide us with their contact details?

End: Is there anything else you would like to share with us?
