# Supplementary material for: Lessons learned from Evidence-Informed Decision-Making in Nutrition & Health (EVIDENT) in Africa: a project evaluation
Source: Health Res Policy Syst. 2019 Jan 31;17:12. doi: 10.1186/s12961-019-0413-6 (PMC6357392; doi:10.1186/s12961-019-0413-6)
Supplement: Supplementary file 3 — Semi-structured questioning route for group II. A questioning route used to guide the in-depth interviews of group II participants. (DOCX 35 kb) [file 12961_2019_413_MOESM3_ESM.docx]

**Additional file** 3 Semi-structured questioning route for group II

Question in yellow = question not to be asked when interviewee is no longer involved in EVIDENT

*General probes:*

- *SILENCE*
- *Can you tell me more about that?*
- *Can you give me an example of that?*

| **Components** | | | **Relevant Evaluation Questions** |
| --- | --- | --- | --- |
| **Description of participant** | | | |
| **Involvement in EVIDENT** | 1. Could you tell me about how you became involved with EVIDENT? 2. What was your motivation to become and (not) stay involved in EVIDENT?   *Probe: for your own benefit?,for the benefit of the EVIDENT process?* | | |
| **Strengthening capacity & leadership** | | | |
| **Capacity** | EVIDENT carried out a number of trainings to strengthen its partners’ capacity in the process of EIDM.   1. Did you participate in these trainings? Or were you involved in another way? (in order to choose A, B or skip question 3) 2. For interviewees who participated in the training:   What was the value of the trainings for you, in performing the work that was required within EVIDENT?  *Probe: personal/professional; skillset obtained?*   1. For interviewees who assisted the training:   What was the value of the trainings for the work that was required within EVIDENT?   1. Beyond this training, what other types of capacity building exercises would have been useful within EVIDENT?   ***!! Probe****: How could capacity building be improved within EVIDENT?* | | |
| **Leadership** | Applicable to coaches:  EVIDENT demanded leadership from its partners in order to achieve its objectives.   1. How has EVIDENT enhanced your own leadership skills? 2. What leadership emerged amongst partners or other actors? 3. How could leadership capacities have been enhanced more amongst partners? | | |
| **Problem-oriented and Evidence-informed Decision-making** | | | |
| **Steps in the conceptual framework:**   1. **stakeholder involvement** 2. **prioritisation of research questions** 3. **evidence products generation** 4. **evidence products contextualisation** 5. **facilitation of evidence** | | **Conceptual framework:**  EVIDENT has developed a conceptual framework to achieve its objectives. I would like to outline this conceptual framework again for you…  This framework is based on the following 3 activity pillars: i) Problem-oriented and EIDM, ii) capacity strengthening and leadership and iii) horizontal collaboration. from mapping stakeholders to the facilitation of evidence. A first step in the framework is stakeholder involvement and the next prioritizing research questions; a second step is generating evidence using high quality evidence synthesis products such as systematic reviews; a third step includes the translation of evidence to the country-specific context; a fourth step is the generation of policy recommendations tailored to the specific country context and starting a policy dialogue which allows stakeholders to make decisions about how to best intervene in their context.   1. What are your views on this conceptual framework for EIDM?   *Probe: What have been your experiences (positive and negative) regarding each step in the framework (stakeholder involvement, prioritisation of research questions, evidence products generation, evidence products contextualisation, facilitation of evidence)?*   1. Could you suggest improvements for the conceptual framework? | |
|  |  | **Tools and process notes:**  / | |
| **Case country studies** | | / | |
| **Horizontal Collaboration/Network** | | | |
| **Communication** | | EVIDENT developed a communication strategy to streamline communication within the collaboration and to increase its visibility.  / | |
| **Visibility** | | 1. What are your views on EVIDENT’s visibility? 2. How could EVIDENT’s visibility be improved? | |
| **Network** | | 1. What has been the added value of EVIDENT operating as a network of partners from North-South, South-south, in-country? 2. Could you suggest a better format for EVIDENT, other than a network of partners?   *Probe: e.g. institution, …* | |
| **MANAGEMENT** | | | |
| **Communication** | | 1. How did you perceive the management’s communication towards partners and also towards external stakeholders such as funding agencies, the SUN movement, IFPRI and others? *(interviewee can be a stakeholder)* | |
| **Operationalisation** | | EVIDENT has an outlined management structure including different bodies like the expert panel (coaches), the coordination body, the country teams, etc.   1. What are your views on the advantages and disadvantages of this overall structure? 2. What are your views on the functioning of EVIDENT’s coordination body?   *Probe: pros and cons of the management structure?* | |
| **Financial** | | */* | |
| **SUSTAINABILITY** | | | |
| Many initiatives like EVIDENT have failed to reach sustainability (e.g. SURE). EVIDENT had as an objective to become a sustainable project at the end of the current seed funding.   1. What should be done to increase EVIDENT’s sustainability?   *Probe: what are the elements needed to make EVIDENT a leading institution in the EIDM process?*   1. Why do you think EVIDENT should continue to exist? | | | |
| **LESSONS LEARNED (barriers, drivers, opportunities, enabling environment)** | | | |
| EVIDENT is ongoing for almost 3 years now. EVIDENT would have experienced success but also faced a number of challenges over the course of three years.   1. What are the lessons you have learned from working with EVIDENT? 2. What could be EVIDENT’s biggest strengths? 3. What could be EVIDENT’s weaknesses? | | | |
| **UNINTENDED CONSEQUENCES** | | | |
| EVIDENT had set out its goals from the beginning, but developed in a very dynamic manner, take for example the kick-off meeting in which the management structure was decided by all partners; so next to the foreseen activities also other things could have taken place.  / | | | |

We would like to reach a snowballing effect in this evaluation in which participants name other people who might be related to EVIDENT in their setting. So could you please name 2 or 3 other stakeholders that have been associated with EVIDENT in your setting? Would you be willing to provide us with their contact details?

End: Is there anything else you would like to tell me?
