# Supplementary material for: Lessons learned from Evidence-Informed Decision-Making in Nutrition & Health (EVIDENT) in Africa: a project evaluation
Source: Health Res Policy Syst. 2019 Jan 31;17:12. doi: 10.1186/s12961-019-0413-6 (PMC6357392; doi:10.1186/s12961-019-0413-6)
Supplement: Supplementary file 4 — Online survey questions for groups I and II. Questions online survey that sent to directly involved participants (groups I and II). (PDF 608 kb) [file 12961_2019_413_MOESM4_ESM.pdf]

Please answer ALL questions.

1. What is your name?

2. What is your gender?

- Female
- Male

3. What is your nationality? (e.g. British, Egyptian, American, etc)

4. What is your educational background? (e.g. BSc in Biomedical Sciences, MSc in Nutrition and PhD in Bioengineering)

5. Where do you work? Please fill in the full address.

Unit and/or Department (e.g. Nutrition Unit, Department of Public Health)

Institution (e.g. Institute of Tropical Medicine)

Address (e.g. 155 Nationalestraat, 2000 Antwerp)

Country (e.g. Belgium)

Telephone No. (+32 3 345 55 55)

6. What is your position or job title at your institution? (e.g. researcher, lecturer, professor, etc)

7. How long have you been at your current position? (Months & Years)

Month(s)

Year(s)

Please answer ALL questions.

8. When did you become involved with EVIDENT? (Month & Year) (e.g. January 2014, July 2015, etc)

Month

Year

9. Are you currently involved with EVIDENT?

- Yes
- No

Please answer ALL questions.

10. What is your current role within EVIDENT?

- [ ] Active Partner
- [ ] Passive Partner
- [ ] Coach/Mentor
- [ ] Scientific Committee

Any other role (please specify):

11. How would you grade the extent of your current involvement in EVIDENT?

Not at all	Very little	Average	Fair	To a great extent
○	○	○	○	○

Please answer ALL questions.

12. When did your involvement with EVIDENT come to an end? (Month & Year) (e.g. January 2014, July 2015, etc)

13. What was your previous role within EVIDENT?

- [ ] Active Partner
- [ ] Passive Partner
- [ ] Coach/Mentor
- [ ] Scientific Committee

Any other role (please specify):

14. Did you receive financial remuneration for your participation?

- ( ) Yes
- ( ) No
- ( ) Don't know

Please answer ALL questions.

15. What type of financial remuneration did you receive for your participation?

- [ ] Full-time Paid Work (including self-employed)
- [ ] Part-time Paid Work (including self-employed)
- [ ] Project Topping-Up
- [ ] Government or Other Training Scheme
- [ ] Temporary Sick
- [ ] Long-term Sick
- [ ] Full-time Education/Masters Student/PhD Student
- [ ] Don't know

Any other type of financial remuneration (please specify):

16. Was the funding adequate to perform the described and/or designated tasks?

- ○ Yes
- ○ No
- ○ Don't know
- ○ Any other answers to this question (please specify):

Please answer ALL questions.

17. EVIDENT carried out a number of trainings to strengthen its partners' capacity in evidence-informed decision-making. Were you a coach/mentor on any of the trainings given by the network?

- ◯ Yes
- ◯ No

18. Which of the following trainings were you a coach on?

- [ ] International Course in Evidence-informed Nutrition (ICEN) (Belgium, September 2014)
- [ ] International Course in Evidence-informed Decision-making for Nutrition and Health (ICENH) (South Africa, May 2015)
- [ ] Specialised Training in Translating Evidence in Nutrition into Country-specific Recommendations (Ethiopia, November 2015)

19. Did you receive any training within the EVIDENT network?

- ( ) Yes
- ( ) No

Please answer ALL questions.

20. Which of the following trainings did you receive?

- ☐ International Course in Evidence-informed Nutrition (ICEN) (Belgium, September 2014)
- ☐ International Course in Evidence-informed Decision-making for Nutrition and Health (ICENH) (South Africa, May 2015)
- ☐ Specialised Training in Translating Evidence in Nutrition into Country-specific Recommendations (Ethiopia, November 2015)

21. To what extent did you value the training(s)?

Not at all	Slightly	Moderately	Very much	Extremely
○	○	○	○	○

22. To what extent did the training(s) contribute to your work within EVIDENT?

Not at all	Slightly	Moderately	Very much	Extremely
○	○	○	○	○

23. To what extent has EVIDENT contributed to strengthening your own capacity?

Not at all	Slightly	Moderately	Very much	Extremely
○	○	○	○	○

24. To what extent has EVIDENT contributed to strengthening the capacity of your local institution/organisation?

Not at all	Slightly	Moderately	Very much	Extremely
○	○	○	○	○

25. EVIDENT demanded leadership from its partners and other stakeholders in order to achieve its objectives. Has EVIDENT enhanced your own leadership skills?

- ○ Yes
- ○ No

26. How much do you agree or disagree with the following statements?

	Strongly disagree	Disagree	Neither	Agree	Strongly agree	N/A
I feel I am listened to when I speak up at partner meetings	◯	◯	◯	◯	◯	◯
I feel in control of my own work within EVIDENT	◯	◯	◯	◯	◯	◯
EVIDENT contributes to my scientific portfolio	◯	◯	◯	◯	◯	◯
I feel at ease with partners to discuss issues	◯	◯	◯	◯	◯	◯

Other (please specify):

Please answer ALL questions.

27. Were you a coach on any of the case studies (including systematic reviews)?

- Yes
- No

28. Which of the following case study or studies were you a coach on?

- [ ] Benin
- [ ] Ethiopia
- [ ] Ghana
- [ ] South Africa

29. To what extent do you think you contributed to the case country study(-ies) as a coach?

Not at all	Very little	Somewhat	To a great extent	Fully
○	○	○	○	○

30. Were you involved in carrying out a case country study (including systematic reviews)?

- ◯ Yes
- ◯ No

Please answer ALL questions.

31. EVIDENT aimed to develop and/or use a series of guidelines to facilitate the stepwise process of evidence-informed decision-making. Were you aware of any of the following guidelines?

	Yes	No
Stakeholder mapping	◯	◯
Prioritisation of research questions	◯	◯
SeCURE tool	◯	◯
Publications & conference abstracts	◯	◯
Quality assurance for policy briefs	◯	◯

32. Did you use the guidelines on any of the following topics?

	Yes	No	N/A
Stakeholder mapping	◯	◯	◯
Prioritisation of research questions	◯	◯	◯
SeCURE tool	◯	◯	◯
Publications & conference abstracts	◯	◯	◯
Quality assurance for policy briefs	◯	◯	◯

33. Were any of the following guidelines helpful?

	Yes	No	Don't know	N/A
Stakeholder mapping	◯	◯	◯	◯
Prioritisation of research questions	◯	◯	◯	◯
SeCURE tool	◯	◯	◯	◯
Publications & conference abstracts	◯	◯	◯	◯
Quality assurance for policy briefs	◯	◯	◯	◯

34. How difficult was it to...

	Very difficult	Difficult	Neither	Easy	Very easy	Did not do this
...identify and engage with stakeholders?	○	○	○	○	○	○
...obtain prioritise research questions?	○	○	○	○	○	○
...generate evidence products (e.g. systematic reviews, policy briefs, etc)?	○	○	○	○	○	○
...contextualise findings from the systematic review?	○	○	○	○	○	○

Any comments (please specify):

35. How actively do you think external stakeholders were engaged with EVIDENT?

Not at all	A little	Somewhat	To a great extent	Fully active	N/A
○	○	○	○	○	○

Please answer ALL questions.

36. Which outputs did your case country study generate based on the initial protocol of EVIDENT?

- [ ] Systematic review(s)
- [ ] Mapped stakeholders
- [ ] Identification and description of the barriers and limitations for evidence utilization in decision making in nutrition
- [ ] Development of a framework for how to integrate evidence to inform priorities and decisions in nutrition policy and programs
- [ ] Publication(s)
- [ ] Policy brief(s)
- [ ] Don't know
- [ ] None
- [ ] Any others (please specify):

37. To what extent did your country team complete the generation of these outputs?

Not at all	Very little	Somewhat	To a great extent	Fully complete	Don't know
○	○	○	○	○	○

38. How many conference abstracts did your country team produce?

39. How many drafts for publications did your country team produce?

40. How satisfied were you with the people and cooperation within your country team?

Very dissatisfied	Dissatisfied	Neither dissatisfied nor satisfied	Satisfied	Very satisfied
○	○	○	○	○

41. How aware were you of the ongoing activities in other case study countries?

Not at all	Very little	Somewhat	To a great extent	Completely
○	○	○	○	○

42. Do you think your country team needed more expertise?

Strongly disagree	Disagree	Neither agree nor disagree	Agree	Strongly agree
◯	◯	◯	◯	◯

43. Was the number of team members in your case country study sufficient to carry out the designated tasks?

Strongly disagree	Disagree	Neither agree nor disagree	Agree	Strongly agree
◯	◯	◯	◯	◯

44. How would you rate the quality of your country team in the following elements?

	Very low quality	Low quality	Neither low nor high quality	High quality	Very high quality
Active participation	◯	◯	◯	◯	◯
Collaboration with the EVIDENT coordination body	◯	◯	◯	◯	◯
Broadening of the EVIDENT network	◯	◯	◯	◯	◯
Achieving the intended outputs	◯	◯	◯	◯	◯
Taking initiative in achieving outputs	◯	◯	◯	◯	◯

45. How satisfied were you with EVIDENT's communication on each of the following elements?

	Very dissatisfied	Dissatisfied	Neither dissatisfied or satisfied	Satisfied	Very satisfied
Conferences	◯	◯	◯	◯	◯
Emails, conference calls, instant messaging	◯	◯	◯	◯	◯
Trainings	◯	◯	◯	◯	◯
Partner Meetings	◯	◯	◯	◯	◯
Website	◯	◯	◯	◯	◯

Any other thoughts (please specify):

Please answer ALL questions.

46. How well do you think EVIDENT's attempts at increasing its visibility have worked?

Not at all	Poorly	Neither	Fairly well	Extremely well	Don't know
○	○	○	○	○	○

47. Thinking about how often you personally contact your colleagues from EVIDENT at other institutes - how often do you do any of the following?

	Never	Rarely	Sometimes	Often	Very often	N/A
Meet up with colleagues	○	○	○	○	○	○
Speak to colleagues	○	○	○	○	○	○
Write to colleagues	○	○	○	○	○	○

Any other comments (please specify):

48. How often did you collaborate (receive or give help) with other EVIDENT colleagues at other institutes?

Never	Rarely	Sometimes	Often	Very often	N/A
○	○	○	○	○	○

Any other comments (please specify):

49. How satisfied were you with EVIDENT's management structure? (The management structure refers to different bodies such as the coaches, coordination body, case country teams and scientific committee)

Very dissatisfied	Dissatisfied	Neither dissatisfied or satisfied	Satisfied	Very satisfied	Don't know
○	○	○	○	○	○

50. How satisfied were you with the communication from the coordination body?

Very dissatisfied	Dissatisfied	Neither dissatisfied nor satisfied	Satisfied	Very satisfied
○	○	○	○	○

51. How satisfied were you with each of the following elements?

	Very dissatisfied	Dissatisfied	Neither dissatisfied or satisfied	Satisfied	Very satisfied
The way you were kept informed about things that might have affected you	◯	◯	◯	◯	◯
The coordination body's willingness to take account of your views when making decisions	◯	◯	◯	◯	◯
The coordination body's accomplishments and way of working	◯	◯	◯	◯	◯

52. How would you rate the degree of transparency from the coordination body?

Very poor	Poor	Acceptable	Good	Very good	N/A
◯	◯	◯	◯	◯	◯

Please answer ALL questions.

53. How would you rate EVIDENT's dynamic approach to making changes and improvements in its processes?

Very poor	Poor	Fair	Good	Very good	Don't know
○	○	○	○	○	○

54. How much did EVIDENT interfere with your normal work (including work for your institute and/or consultancies)?

A lot	Quite a bit	Somewhat	A little	Not at all
○	○	○	○	○

55. Think about the work that you were supposed to deliver within EVIDENT. Which of the following statements apply to you?

○ Accomplished what I was responsible for

○ Accomplished less than I would have liked to have

○ Accomplished more than I was responsible for

56. Which of the following barriers would apply to you?

☐ My work commitments were overwhelming and prevented my active participation in EVIDENT

☐ I had no and/or inadequate institutional support

☐ My country team was too small

☐ I had insufficient funding

Any other barriers (please specify):

57. How satisfied are you with your work in EVIDENT?

Very dissatisfied	Dissatisfied	Neither satisfied nor dissatisfied	Satisfied	Very satisfied
○	○	○	○	○

58. How was your experience in working with EVIDENT?

Very poor	Poor	Average	Good	Very good
○	○	○	○	○

Please elaborate further:

59. To what extent do you agree with the following statements?

	Strongly disagree	Disagree	Neither agree not disagree	Agree	Strongly agree	N/A
Being a part of EVIDENT makes me highly valued	○	○	○	○	○	○
Stakeholders think EVIDENT has a good reputation	○	○	○	○	○	○
People in my institute think highly of EVIDENT	○	○	○	○	○	○
Alone, or with others, I can influence decisions affecting my local setting	○	○	○	○	○	○

60. When thinking about continuing EVIDENT's work, which of the following statements sums up your views on EVIDENT?

- ☐ EVIDENT is fine as it is in terms of sustainability
- ☐ EVIDENT needs some work to improve its sustainability
- ☐ EVIDENT needs major work to improve its sustainability
- ☐ EVIDENT has no added value, so it does not need to be sustainable
- ☐ Don't know
- ☐ N/A
- ☐ Any other statements (please specify):

61. What measures were taken to try to increase and/or ensure EVIDENT's sustainability?

- [ ] Finding further funding
- [ ] Handing over the project to a southern partner
- [ ] Building the EVIDENT network further
- [ ] Extending the duration of the project
- [ ] Don't know
- [ ] N/A
- [ ] Any other measures (please specify):
