# Supplementary material for: Lessons learned from Evidence-Informed Decision-Making in Nutrition & Health (EVIDENT) in Africa: a project evaluation
Source: Health Res Policy Syst. 2019 Jan 31;17:12. doi: 10.1186/s12961-019-0413-6 (PMC6357392; doi:10.1186/s12961-019-0413-6)
Supplement: Supplementary file 5 — Online survey questions for group III. Questions from the shorter online survey that was sent to indirectly involved participants (group III) (PDF 105 kb) [file 12961_2019_413_MOESM5_ESM.pdf]

Please answer ALL questions.

1. What is your name?

2. What is your gender?

- Female
- Male

3. What is your nationality? (e.g. British, Egyptian, American, etc)

4. What is your educational background? (e.g. BSc in Biomedical Sciences, MSc in Nutrition and PhD in Bioengineering)

5. Where do you work? Please fill in the full address.

Unit and/or Department (e.g. Nutrition Unit, Department of Public Health)

Institution (e.g. Institute of Tropical Medicine)

Address (e.g. 155 Nationalestraat, 2000 Antwerp)

Country (e.g. Belgium)

Telephone No. (+32 3 345 55 55)

6. What is your position or job title at your institution? (e.g. researcher, lecturer, professor, etc)

7. How long have you been at your current position? (Months & Years)

Month(s)

Year(s)

8. Are you associated with the Scaling Up Nutrition (SUN) Movement, either personally or through your institution and/or projects?

- Yes
- No

Please answer ALL questions.

9. How are you associated with the SUN Movement? (e.g. consultant for SUN Movement, speaker at SUN Global Gathering 2015, etc)

10. When did you become involved with EVIDENT? (Month & Year) (e.g. January 2014, July 2015, etc)

Month

Year

11. Are you currently involved with EVIDENT?

- Yes
- No

Please answer ALL questions.

12. How are you involved with EVIDENT? (e.g. participating in a conference together, conducting a systematic review through EVIDENT, etc)

13. What are your reasons for becoming involved with EVIDENT?

14. How has your experience been in working with EVIDENT?

Very poor	Below average	Average	Above average	Excellent
○	○	○	○	○

Please elaborate further.

Please answer ALL questions.

15. When did your involvement with EVIDENT come to an end? (Month & Year) (e.g. January 2014, July 2015, etc)

16. How were you involved with EVIDENT? (e.g. participating in a conference together, conducting a systematic review through EVIDENT, etc)

17. What were your reasons for becoming involved with EVIDENT?

18. How was your experience in working with EVIDENT?

Very poor	Below average	Average	Above average	Excellent
○	○	○	○	○

Please elaborate further.

19. Did you receive financial remuneration for your participation?

- ○ Yes
- ○ No
- ○ Don't know

Please answer ALL questions.

20. What type of financial remuneration did you receive for your participation?

- [ ] Full-time Paid Work (including self-employed)
- [ ] Part-time Paid Work (including self-employed)
- [ ] Project Topping-Up
- [ ] Government or Other Training Scheme
- [ ] Temporary Sick
- [ ] Long-term Sick
- [ ] Full-time Education/Masters Student/PhD Student
- [ ] Don't know

Any other type of financial remuneration (please specify):

21. Was the funding adequate to perform the described and/or designated tasks?

- ○ Yes
- ○ No
- ○ Don't know
- ○ Any other answers to this question (please specify):

Please answer ALL questions.

22. How would you rate EVIDENT's visibility?

Very poor	Poor	Fair	Good	Very good	Don't know
○	○	○	○	○	○

23. What are your views on EVIDENT's visibility?

24. How do you think EVIDENT can improve both its visibility and communication?

25. Do you think EVIDENT should continue to exist?

- ○ Yes
- ○ No

Please answer ALL questions.

26. Why do you think EVIDENT should continue to exist?

27. How can EVIDENT ensure its sustainability? What needs to be done by EVIDENT in order to ensure its sustainability?

28. How do you think EVIDENT can be improved on the whole?

29. Would you like to add any further thoughts on EVIDENT?
