# Supplementary material for: Lessons learned from Evidence-Informed Decision-Making in Nutrition & Health (EVIDENT) in Africa: a project evaluation
Source: Health Res Policy Syst. 2019 Jan 31;17:12. doi: 10.1186/s12961-019-0413-6 (PMC6357392; doi:10.1186/s12961-019-0413-6)
Supplement: Supplementary file 6 — Node tree for the analysis of in-depth interviews. A node tree of code, analyse and summarise in-depth interviews (DOCX 899 kb) [file 12961_2019_413_MOESM6_ESM.docx]

# Additional file 6 Node tree for the analysis of in-depth interviews

# *
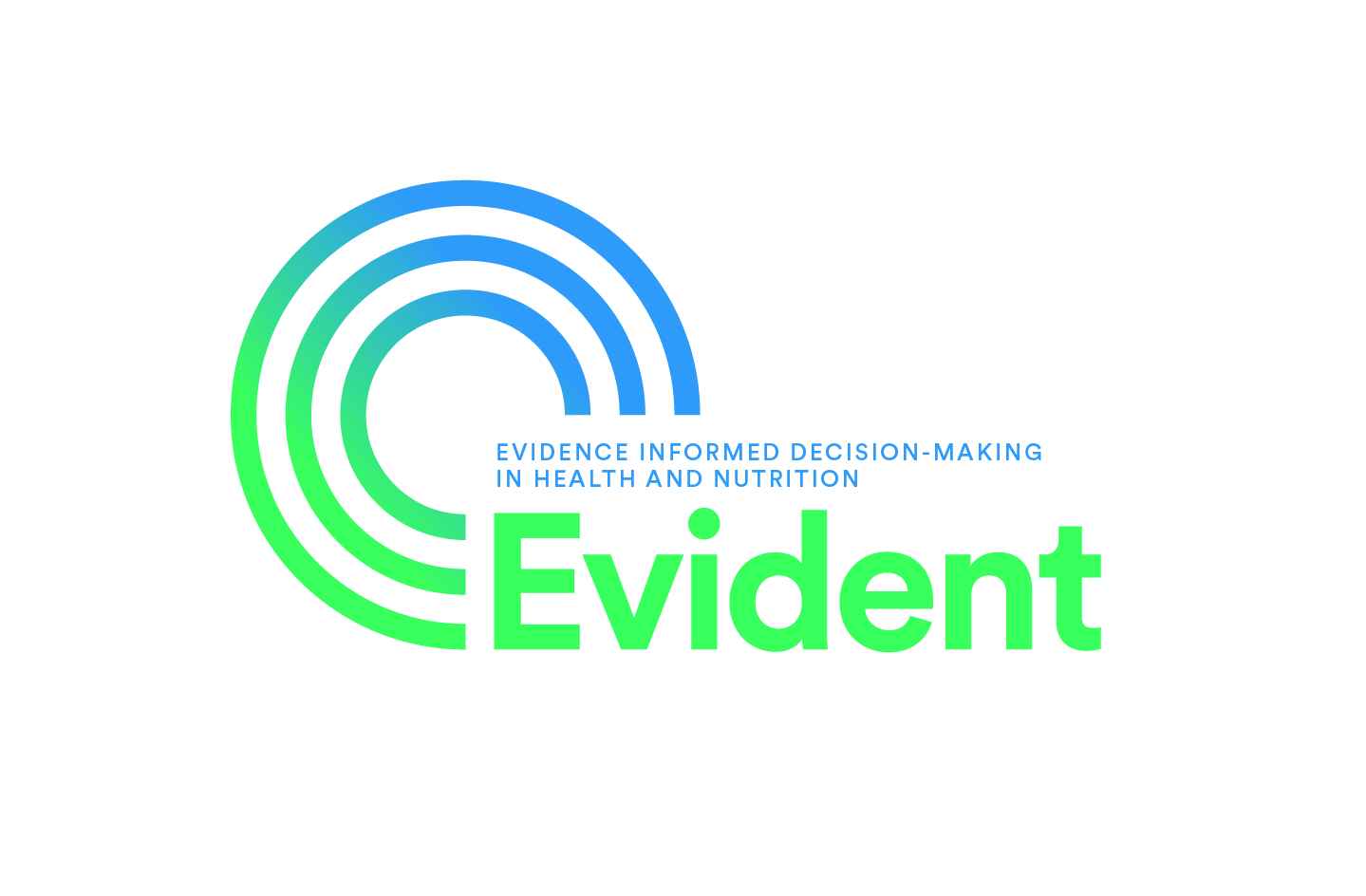
*
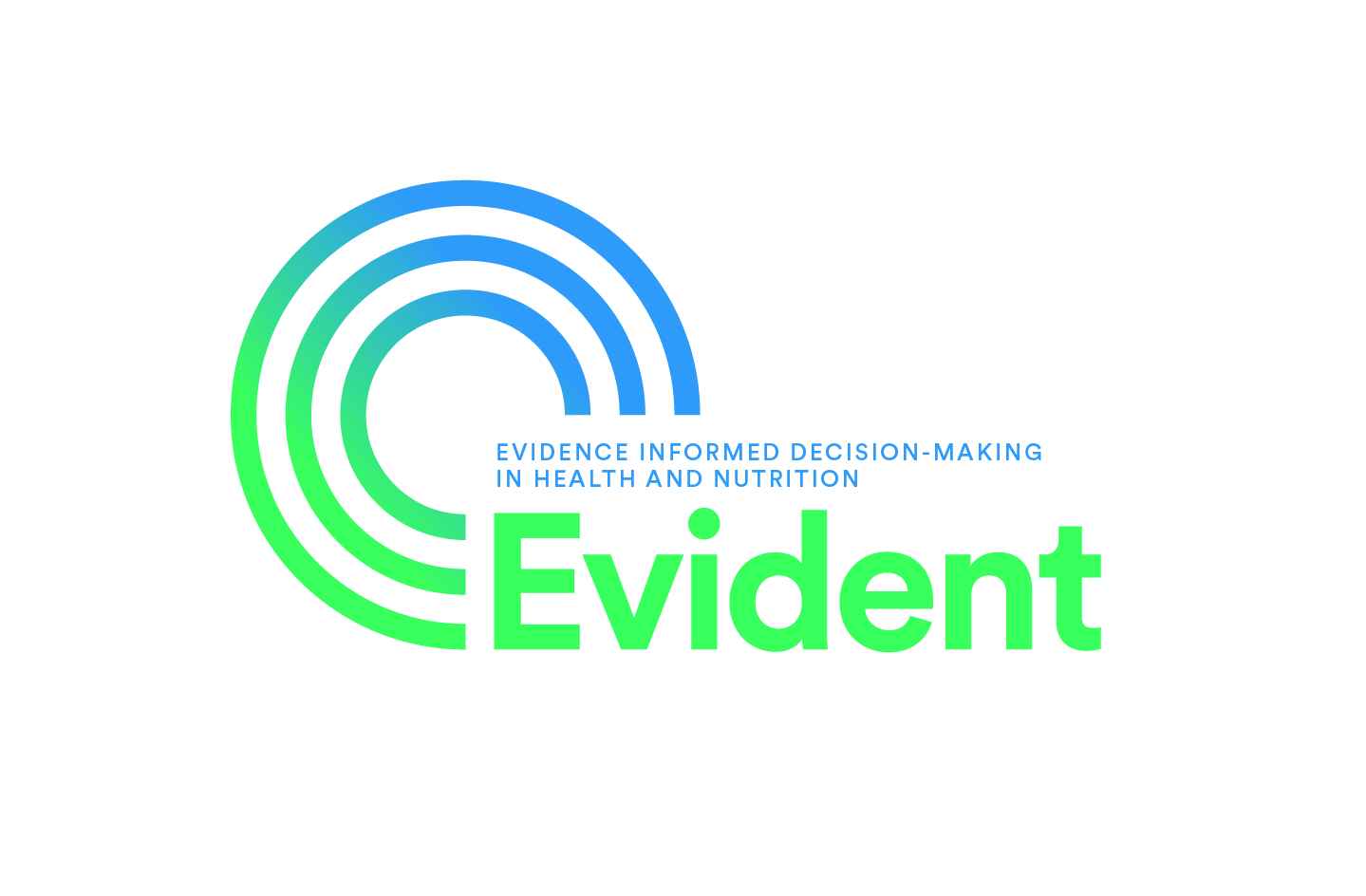


1. **CAPACITY & LEADERSHIP**

*Capacity is the capability of doing your work (within EVIDENT). Capacity building is going through the necessary processes/teaching/learning in order to increase your skills in order to perform your work. This node would take into account all quotes about EVIDENT’s capacity strengthening/building exercises such as the trainings.*

*Leadership is the activity of leading a group of people or an organization or the ability to do this. It holds: establishing a clear vision, sharing that vision with others so that they will follow willingly, providing the information, knowledge and methods to realize that vision, and coordinating and balancing the conflicting interests of all members and stakeholders.*

- 1. **Capacity building**

*What is the added value of capacity building within EVIDENT?*

- - 1. **Attitude towards skill development**

*Attitude towards acquiring capacity and/or technical skills to carry out the stepwise EIDM process, outside of the trainings*

- - 1. **Perception of processes behind EIDM**

*Perception of the processes that go behind EIDM as understood from the trainings and capacity building exercises.*

- - 1. **Training**

*Learnings from the courses carried out by EVIDENT. What have you learned from the courses? What was the value of the training?*

- 1. **Leadership**
     1. **Perception of Leadership**

*Views on leadership as a general topic. Changes/views in how partners understand/appreciate leadership. This would also include barriers, opportunities and enabling environment on leadership*

- - - 1. **Individual leadership**

*Did the leadership skills of the participant increase through the EVIDENT project? Experiences of personal leadership development.*

- - - 1. **At Country Level**

*Leadership at country level could be broader than the country team and also focuses on the interaction with other stakeholders. Moreover, did leadership emerge amongst partners in their country teams, in their setting?*

- - - 1. **Within EVIDENT**

*Did leadership processes emerge amongst partners, within the EVIDENT project as a whole? Did an individual person(s) take the lead? (This would include quotes about individual bearing most leadership)*

- - 1. **Awareness of leadership**

*Becoming more aware about leadership, the need for it and/or its importance, as a whole*

1. **PROBLEM ORIENTED and EIDM**
   1. **Operationalisation**

*Looking at the following topics in terms of their operationalisation/functionality:*

- - 1. **Conceptual framework**

*Conceptual framework as developed during the project, with the 3 main pillars.*

- - - 1. **Perception of framework**

*How is the conceptual framework model seen? What is the general view on the conceptual framework (some participants commented generally, not on each step in the framework). Views of the different steps in the conceptual framework.*

- - - 1. **Perception of the different tools**

*What are the views on the different guidelines, process notes, tools (to facilitate stakeholder mapping, prioritisation of a research question, evidence synthesis products such as systematic review, policy brief, and publications)*

- - 1. **Attitude towards project format**

*Should EVIDENT operate as anything other than a network?*

- 1. **Problem-oriented and EIDM**
     1. **Case Country Study level**
        1. **Experience with EIDM Processes**

*Experiences working within (as a ccs member) or working with (as a coach, mentor) a case country study, learnings and added values from this experience.*

- - - 1. **Perception of the EIDM Processes**

*Perception of the processes that go behind EIDM as understood from the case country study. How is the EIDM process perceived, as understood from the ccs?*

- - - 1. **Attitude towards EIDM Processes**

*Feelings towards the EIDM Process as understood from the case country study*

- - - - 1. **Attitude towards stakeholder engagement**

*Feelings towards engaging with stakeholder, as part of the EIDM process*

- - - - 1. **Attitude towards contextualisation**

*Feelings towards the contextualisation process. EIDM processes to the local context. Also includes the views on (the need for) adaptation of the conceptual framework to real life contexts*

- - 1. **Overall EVIDENT level**
       1. **Experience with EIDM Processes**

*Experiences working within EVIDENT on EIDM processes, learnings and added values from this experience.*

- - - 1. **Perception of EIDM Processes**

*Insight into the decision-making processes overall. Views on how EIDM should be done generally, new insights or opportunities in terms of future EIDM processes. This is does not include Perception of EIDM Processes from CCS.*

- - - 1. **Attitude towards EIDM Processes**

*Feelings towards the EIDM Processes overall, not from carrying out the CCS*

- - - - 1. **Attitude towards stakeholder engagement**

*Feelings towards engaging with stakeholders outside of the ccs*

1. **HORIZONTAL COLLABORATION**

*EVIDENT was established as a network, and involved horizontal collaboration between partners and with other stakeholders.*

- 1. **Perception of network values**

*Added values of a network of partners:*

- - 1. **Knowledge sharing**

*The network allowed partners to share information, learnings and experience amongst themselves*

- 1. **Network functionality**

*To what extent did the network work/was operational and was spread/was known in the country setting of the participant, in N vs S or S-S.*

- 1. **Network interactions**
     1. **Within the network**

*For example interaction between partners or between coaches and case country study members*

- - 1. **With other stakeholders**

*Did partners initiate, develop or establish collaborations with other stakeholders of EVIDENT? How did the network relate to other stakeholders?*

- - 1. **S-S/N-S distortions**

*Differences in perceptions between North and South on project matters and visions on the future. Includes also imbalances in the uniformity of collaborations . Differences in views/engagement on the project between N and S, and even S-S.*

1. **MANAGEMENT**

*Management structure with different bodies (coordination body, scientific committee, coaches, country teams)*

- 1. **Communication**
     1. **Perception of communication**

*Views on the communication within the network in general. communication strategy, which was developed as a work package.*

- - - 1. **Within EVIDENT**
      2. **Towards external stakeholders**
    1. **Attitudes towards communication medium**

*Views on the means used to communicate such as face to face meetings, emails etc.*

- - 1. **Perception of transparency**

*Views on how transparent the communication was within the network?*

- 1. **Management Structure**
     1. **Attitude towards Management Structure**

*Feelings towards the management structure as a whole (which includes the different bodies: coordination body, case country studies, caches, scientific committee). This also includes views on the utilisation of this structure and how this could be improved.*

- - - 1. **Attitude towards coordination body**

*Feelings specifically towards the coordination body, which entails the managing partners at ITM*

- - - 1. **Attitude towards administrative functionality**

*Feelings towards the functionality of the administration level: procedure, etc.*

- 1. **Visibility**

*Visibility is the extent to which evident was visible/known/broadcasted/spread to the general public, to stakeholders, to people within the nutrition field, to other fields etc.*

- - 1. **Perception of project visibility**

*Views and thoughts on how visible the project is, how visibility developed.*

1. **OUTPUTS**
   1. **Perception of planned outputs**

*Views on the outputs that were expected initially from the project, such as systematic reviews, policy briefs, publications. Views on the extent in which these outputs were reached or not reached.*

- 1. **Perception of unexpected outputs**

*Views on the outputs that were not expected initially from the project but nevertheless evolved during the project.*

1. **SUSTAINABILITY**
   1. **Perception of project sustainability**

*Perceptions on what was done to ensure sustainability, the extent in which the project is sustainable and on what is needed to be done more.*

- 1. **Perception of local ownership**

*Views on the initiation of local ownership in the project.*

1. **LESSONS LEARNED**

*This includes all the lessons that were learned during the evaluation, by participants during the project etc in terms of barriers, drivers, enabling environment and opportunities. Moreover, what re the important elements of the project or the people to become and stay involved in EVIDENT.*

- 1. **Barriers**

*Elements that held back/challenged/limited the project or certain facets of the project.*

- - 1. **Time**

*Barriers in terms of time needed to carry out EVIDENT activities*

- - 1. **Funding**

*Barriers in terms of not enough funding to carry out EVIDENT activities, level of difficulty getting hold further of funding*

- - 1. **Distance**

*Barriers in terms of location, being distant from each other.*

- - 1. **Experience in EIDM**

*Barriers in terms of lack of experience in EIDM which hindered the progress of the project*

- - 1. **Challenging environment**

*External factors that may influence EIDM in country settings*

- 1. **Drivers**

*Elements that facilitate the project or certain facets of the project in moving forward*

- - 1. **Personal interest**

*Drivers in terms of personal interest in a topic or the project*

- - 1. **Commitment**

*Drivers in terms of committing to the project or wanting to contribute to the project.*

- - 1. **Local needs**

*Drivers in terms of local needs, needs for EIDM*

- 1. **Opportunities**

*An opportunity that EVIDENT created for the individual or institute throughout the duration of the project*

- - 1. **Career opportunity**

*Career opportunities offered by the project, extending opportunities to have an impact on something*

- - 1. **Networking opportunity**

*What opportunities were offered by the network to interact with others in the same field?*

- - 1. **Training opportunities**

*Opportunities offered by EVIDENT with regards to capacity building through training.*

- 1. **Enabling Environment**

*Existing factors in the environment that facilitated the project to advance*

- - 1. **Internal**
       1. **Trustworthy partnership**

*Enabling environment in terms of having/building trustworthy partnerships*

- - - 1. **Funding**

*Enabling environment in terms of (sufficient) funding for the project*

- - 1. **External**
       1. **Background**

*Enabling environment in terms of an existing setting/background before starting the project which enables the setting up and process of the project*

- - - 1. **Previous Collaborations**

*Drivers in terms of previous networks that were built up (e.g. SUNRAY, SUN), cooperating as a network, colleagues previously being involved in networks/projects*

- 1. **Recommendations**

*Elements that could/should be provided in the future for EVIDENT*

- - 1. **Recommendations for visibility**

*Views and thoughts on how to improve the visibility of the project. Opportunities in terms of feedback for improvement of the visibility.*

- - 1. **Recommendations for Training**

*Views and thoughts on how to improve capacity building through training*

- - 1. **Recommendations for Network Functionality**

*Views and thoughts on how the EVIDENT network should function*

- - 1. **Recommendations for Communication**

*Views and thoughts on how to improve EVIDENT’s communication*

- - 1. **Recommendations for Coordinating Body**

*Views and thoughts on how to improve EVIDENT’s coordination*
