# Supplementary material for: Lessons learned from Evidence-Informed Decision-Making in Nutrition & Health (EVIDENT) in Africa: a project evaluation
Source: Health Res Policy Syst. 2019 Jan 31;17:12. doi: 10.1186/s12961-019-0413-6 (PMC6357392; doi:10.1186/s12961-019-0413-6)
Supplement: Supplementary file 7 — Matrix of supporting data. A matrix illustrating data triangulation from EVIDENT documentation, online surveys, in-depth interviews and the participatory discussion (DOCX 70 kb) [file 12961_2019_413_MOESM7_ESM.docx]

**Additional file 7** Matrix of supporting data

| **Framework element** | **Proving**  Assess the extent to which EVIDENT’s intended activities were implemented and document the lessons learned (drivers, barriers, and opportunities) | **Improving**  Draw on the lessons learned to inform future activities of EVIDENT, and EIDM in nutrition overall |
| --- | --- | --- |
| **Capacity Building** | | |
| **Capacity Building** | **Documentation:**   - 67 individuals trained: 35 in Belgium, 15 in South Africa, and 17 in Ethiopia. This was 22 more than originally intended. - 4 trainings were delivered as planned: 2 took place in Belgium, 1 in South-Africa, and 1 in Ethiopia. - 2 face-to-face modules of 2 weeks each were developed and implemented; modules included systematic reviews, and translating evidence into country-specific recommendations and policy brief development. Modules were based on the expressed needs of partners to address their lack of capacity in specific expertise and to facilitate the completion the case studies in their countries. - 5 follow-up virtual sessions were organised by coaches/experts in evidence synthesis at the University of Sheffield and ITM. - Beninese partners took the initiative to organise two trainings on systematic reviews in their own settings, without financial support. These trainings trained a total of 18 local researchers. | **Interviews*:***  Incorporating evidence synthesis activities into daily work:   - *“so we need training and we also need tools. And we prefer that we get a lot of training here than in the north. In the north just to use the things [tools] that we don’t have in our country and the rest [of the training] continue that in the south.”* - *“I think it can be sustainable by all the institutions adding in evidence synthesis modules into their undergraduate and Master’s programs in nutrition and also then encouraging including dissertation regulations that allow students to do systematic reviews.”* - *“there could be more use of e-learning. But personally, my circumstances don’t always allow me to attend face-to-face courses to deliver, and this was particularly the case with the two EVIDENT courses. The face-to-face model I understand is important for developing relationships between the participants, but I think an alternative would be a greater use of e-learning technologies.”* - *“maybe exchanges. I think we haven’t really explored the idea of exchanging staff. Perhaps having people in our unit working on a few things and also try to understand a little bit how we look at things and how other people look at things.”*   Practical/functional capacities:   - *“you cannot engage with policymakers if you do not have any leadership skills, and I think that is a part that wasn’t incorporated in the training”* - *“I think that they require training around how to generate evidence and how to be able to engage policymakers to, you know, to work together to develop interventions and policy that can change things in their settings. I think, probably, I think also maybe in terms of how do we do that?”* - *“I also think more practical functioning, so to speak, as in umm how to say, giving them the capacity to search online in libraries, giving them access to it, it's very technical capacity but I think that's what we could have invested more in”* - *“… would be useful to have a training for someone in the team about how to manage research, I mean a library”* - *“maybe a little bit of guidance in developing a country team, who should be your country teams”* - *“the challenge is maybe fulfilling every guideline may require a lot of input, a lot of capacity building because even though we may have a standard guideline developed, to fulfill those standards it may be challenging for every country. It requires a lot of capacity building”* |
|  | **Online surveys:**   - Trainings were highly valued in that 7 out of 7 respondents from the online survey rated the extent to which they valued the trainings, which was either ‘very much’ or ‘extremely’. - 7 out of 7 survey respondents felt that on a scale of ‘not at all’ to ‘extremely’ the contribution of the trainings to their own capacity and to their own work has either been ‘very much’ or ‘extremely’. |  |
|  | **Interviews:**  Insight into the importance of EIDM processes:   - *“having done the trainings, it gives you a certain insight in how policies are shaped and what evidence policies actually use”*   *“so we try to really now start seeing and looking at [it] from programme implementer point of view and policy point of view. Are they really understanding what we are talking about? Even if it is not sophisticated statistics, do they really know how to change it to an action or a programme? That is a big change”*  New skills:   - *“it [the trainings] gave me an in-depth knowledge of the sort of questions that the participants were interested in addressing, so it helped to integrate me more in[-to the] EVIDENT project. It also gave me an idea of the current state of knowledge and learning needs which helped me specifically within EVIDENT, but also with my other teaching activities as well”* - *“… I think the value of the training was to open up my scope and then also to learn new skills”*   Value:   - *”it was very essential because I mean one of the main thoughts of the whole EVIDENT concept was…identifying good evidence through systematic reviews, contextualising evidence. I had never done a systematic review** and so if I was going to be involved in this process, it was important that I know how to do this. And so it was very crucial I mean to the whole, the steps of doing evidence-informed decision-making. Very very needed and I thought very important”*   Training approach:   - *“a single in-class training is not sufficient. There should be mentorship and follow-up afterwards. The [in-person] training sessions and the [virtual] follow-up courses held by [the coaches] was really helpful”* - *“I think the development of the two modules and initially we just had one module on systematic reviews and so over time it evolved into two different modules that was based on the needs of the partners at that moment in time.”* - *“Tailoring the training needs and the follow-ups to the needs [of partners]…so this kind of adaptation was good”*   Building different kinds of capacities:   - *“good leadership lessons learned on how to talk to donors and funders”* - *“it has most certainly helped me with dealing with conflicts, because in our own small country team, lots of different personalities, different priorities and different needs, and yeah that makes for conflict. So that’s something that I think worked out well”* - *“Indirectly I suppose, my association with EVIDENT has helped to develop a leadership role in evidence synthesis activities”* - *“the involvement with the northern partners and the interactions with the southern partners helped to establish academic leadership in that way.”* - *“the multi-country, multi-culture, multi-work environment, sort of experience, I think is a huge strength. It is frustrating and it is maybe less effective and efficient and all sorts of things, but having the experience of going through such a process is a huge strength, because it builds different kinds of capacity that many other projects have not built. I think that is really a big strength”*   Time barrier:  *“challenges that have been faced, that are slowing the progress down. Because this is real stuff. It has to do with the [work] environment within which our colleagues have to operate. That’s the [work] environment they must function in on a daily basis. So the issue is ‘how do you work around that?’ as opposed to getting frustrated for the lack of progress”*  Relationship with coaches:   - *“the coaching system has worked well and this is partly because I have had good relationships with the reviews that I’ve been responsible [for]”*: - *“I think big part of the structure was underutilised, for example the scientific committee was very willing and eager to help with moving things forward, having said people were learning how to do the systematic reviews, many countries were still learning. I can speak for Ghana, it took a long while to even request for information because we didn’t know [what] we wanted to request.”* |  |
| **Governance and Leadership** | | |
| **Governance and Leadership** | **Documentation***:*   - The management structure comprised of a coordination body, country teams, a scientific expert panel, and a quality assurance board was set up. Co-ordination body provided leadership and administrative management. Active teams were set up in 4 African countries, leading implementation of the case studies and implementation of evidence synthesis activities. A panel of scientific experts provided training and coaching, and the quality assurance board was contacted to provide quality checks for the reviews. - EVIDENT provided its partners with an opportunity to lead project activities, which evoked personal leadership over time. | **Interviews:**   - *“I think it works if you've got an institution. I think that's been the biggest thing, we sort of set up a management structure for an institutional organisation which EVIDENT isn't and never was. It was a project, it was a network project and so the way it was structured was, in the beginning, a bit ambitious I think for a three-year project.”* - *“we can learn from our errors. For example, not setting regular calls, no matter how we will manage to do this call. And take on the leadership in the south. Clearly defining how it will look like: who is the leader? Who is the deputy leader? And what are the duties? And what other partners will do to help them manage the network?”* - *“I think the groups [case country teams] require leadership skills, how to work in teams to function better. And so as I said earlier, if you don’t have that skill, you don’t have, I mean some of them are not really progressing and when we meet to review we realise that there are some that are really really well-organised, others are not well-organised. You just see that there’s leadership gap in these kind of teams”* - *“positive encouragement and support and helping each other out when it's difficult is very important”* - *“there’s been a lot of space and a lot of room for them to mentor and [to] be mentored so I think yah this is something that we could have pushed you know a bit more to be mentored in or even in, I mean maybe specifically across different countries”* - *“we could have enhanced leadership capacities by really working closer with partners”* - *“Maybe the team could be extended a bit and given small means for communication, for the network of EVIDENT, to call partners and set up virtual calls. And a permanent assistant who is assigned to managing the network and the communication with partners. I mean the team is small. There is a lot to do but the team is small. The same person is travelling, writing proposals, sending emails around, organising gatherings with country partners, like the meeting in Ghana. I mean it’s a lot to do. The team is very small. It needs to be extended”* - “*we can shift from two years or one year [in that] if ITM coordinate overall and then next round other universities might be, some other university maybe UK or somewhere [else] who has [the] capacity to coordinate”* |
|  | **Online survey:**   - 7 of 15 survey participants were ‘satisfied’ with the management structure while 6 ‘were neither dissatisfied nor satisfied’. - 11 out of 16 survey respondents thought the dynamic approach (adaptability) of EVIDENT to making changes and improvements in process was ‘good’. - 11 of the 15 respondents were ‘satisfied’, if not ‘very satisfied’, with the coordination body’s willingness to take into account everyone’s views when making decisions. - 46% of survey respondents however were ‘neither dissatisfied nor satisfied’ or ‘dissatisfied’ with the coordination body’s accomplishments and way of working. - 10 out of 15 survey respondents (1 skipped this question) from the survey felt that EVIDENT enhanced their own leadership skills. - 7 out of 8 participants were ‘satisfied’ or ‘very satisfied’ with the people and coordination within their own country teams; only 1 was ‘very dissatisfied’. - 3 out of 8 survey respondents thought the initiative taken by the country teams to achieve their outputs was of ‘neither low nor high quality’. The remainder 5 out of 8 however considered this initiative to be of either high or very high quality. |  |
|  | **Interviews:**  Overall management structure:   - *“I think it’s a well-organised structure. Yes, you can’t start [without it]. A network like this needs a coordinating body. If you don’t have a coordinating body that really understands the different partners and being able to address their needs then you will not succeed”* - *“the management structure with the expert panels and the country teams. That was really prepared, that was really the Mercedes type of model. We never used that. The advisories were they ever contacted? I don’t know. The country teams, yes that worked. The scientific committee, was that really? It was a very formalised structure, but we never reached a momentum or a volume in the project that really required such a structure. On the other hand it’s good to have those things in place in case one grows rapidly and structures are needed. It was ready but I don’t think we ever needed it”*   Structural flexibility:   - *“[the coordinator] uses the word ‘organic process’, and because it's organic it evolves and it's changing and while that is also as a strength it also presents itself as a weakness because you know there isn't a structure per se, for instance like how we all started at different places”* - *“having a participatory approach sometimes means that people don't necessarily feel a strong sense of direction because it's not top-down direction. So that's the kind of disadvantage of taking the approach to a partnership, I think”*   Co-ordinating body:   - *“ITM sort of had the leadership because the funding was coming through DGD and so it made sense that we [ITM] would coordinate”* - *”the coordination body also worked very well in terms of the admin side of logistics”* - *“I have to lift my hat to [the coordinator]. If it was not for [the coordinator], these things would not work. And again, she had to push everybody everywhere all the time, all the time, all the time. And she will probably never get the recognition that she should have. That she should get from EVIDENT. But yeah, I cannot stress enough, in bold capital letters, if it was not for the effort of [the coordinator] none of it would happen because she was the one who had to deal with these sorts of day to day technical small things, and she had this amazing capacity to absorb a lot of conflict and resistance. And she had to give a lot of energy to get people to do things”* - *“I think it’s fantastic. It’s [the coordinating body has] been able to hold it together. Without the coordination body, EVIDENT wouldn’t exist.”* - *“I think they did a good job of promoting the project internationally, really good job of getting it known, trying to get funding and also communicating with the partners, trying to get partners to take ownership”* - *“we really knew at all times what was going on in terms of where EVIDENT is moving”* - *“I think a lot of the burden fell on [the coordinator] to communicate, and that was a very heavy load, and as a consequence perhaps we had communications that were, sort of, how should I say [it], maybe delayed or not necessarily as clear as they could be”* - *“I think it gave me a good appreciation of seeing somebody else driving something and seeing what the kinds of frustrations are that they...to see the kinds of frustrations that she has to endure to make something like this [EVIDENT] work”… She constantly has to push and pull and this and this and that and that’. And in that sense I think the project, so the leadership, was sort of left to her”*   Country teams and case studies:   - *“I think quite a lot of them [case study teams] have improved their leadership capacities, yes because they all sort of installed a local team that were working with them, had to manage it, had to manage systematic review team and so the skills to do that I think it's where they improve themselves”* - *“I think the advantage is that one, in the country teams it was up to the country. So there was nothing described how we needed to structure ourselves. We relied on our own insight on how to coordinate and structure our team and I think we did that well”* - *“you’re being given a responsibility [case study] and you need to see it through and it’s not a responsibility that you can achieve by yourself, and so it required coming home, organising, collaborating with others with similar interests, and keeping them going, keeping them motivated and following up”* - *“I had too much activities going on at the same time and so you sort of lose, it's an opportunity cost you invest in what have to be done, you set your priorities but also it's at the expense of the network and we could have done much more I think. So also in that sense, it has been a really good lesson in leadership.”* - *“a bigger problem is time allocation. Everybody is very busy…so, I mean, this is for me the most, what I see, this has been the most limiting factor. Everybody is very enthusiastic in saying ‘yes, yes’, but in the end they have other time allocations. They just can’t free themselves enough to advance”* - *“I also see the just engaging with stakeholders or getting hold of stakeholders is a whole mission on its own. So for me that was also really a challenging thing because I had never done that before”* - *“we didn't have enough knowledge about our political system I think. So we had to learn from scratch who is sitting where and who is influencing whom”* - *“it was very difficult to set up a team, a local team here. Because we are French speaking here in Benin and the work has to be done in English: the systematic review, and all the documents that are being reviewed are most of the time written in English. So the person to work on this has to not only be good at English, but also fluent in speaking English, in understanding English. So this has been very difficult for me to set up a team.”* |  |
| **POEIDM – Case Studies** | | |
| **POEIDM – Case Studies** | **Documentation:**   - EVIDENT developed a conceptual framework on the stepwise process of EIDM. - EVIDENT developed 5 process notes on stakeholder mapping, prioritisation of research questions, searching for qualitative research for inclusion in a systematic review, publications and conference abstracts, and quality assurance of policy briefs. - 4 case studies; 1 each in South Africa, Ethiopia, Benin, Ghana. 15 questions identified by case studies and trainings. 4 questions prioritised and addressed using systematic reviews (1 per case country study). 1 additional question addressed by EVIDENT’s Moroccan partner. Institutional Review Board approval received for all reviews. 4 out of 5 systematic reviews in progress with guidance and validation from EVIDENT’s coaches.   Questions:  South Africa: Effective home, family and community based interventions to inform the breastfeeding agenda in South Africa (CRD42016038451),  Ghana: Exclusive breastfeeding duration and child feeding behaviours: A mixed methods systematic review (CRD42016037471)  Benin: Can stunting be reversed after two years of age?  Morocco: To what extend do site-based training, mentoring and operational research improve district health system management and leadership in low- and middle-income countries (CRD13643016023932)  Ethiopia: The effect of community based nutrition intervention on the reduction of stunting and child mortality in under-five years’ children of low and middle-income countries (not registered yet)   - Ethiopia developed a policy brief on the effect of palm oil consumption on health, and a policy brief on exposure of aflatoxin in Ethiopia and measures to be taken. - EVIDENT published in the Annual Trends and Outlook Report 2015, Regional Strategic Analysis and Knowledge Support System chapter. - Blog on the EVIDENT partnership by Michelle Holdsworth posted in the Public Health newsletter of Sheffield University. - EVIDENT is mentioned in the online report published by SUN Movement; 2 country articles of Ethiopia and Ghana in the In Practice Brief issue April 2016. - 2 systematic reviews done by Sheffield students, as an idea that came from working with EVIDENT:   - Oluwabukola Olaitan, Rebecca Pradeilles, Emmanuel Cohen,- Hibbah A. Osei-Kwasi, Michelle Holdsworth (2016) Body size preferences of African women: A mixed-methods systematic review. Obesity Reviews (under review). CRD42015020509 (as part of MPH research attachment, 2014-15)   - Gissing SC, Pradeilles R, Hibbah A. Osei-Kwasi,Emmanuel Cohen, M Holdsworth (2016) Drivers of dietary behaviors in women living in urban Africa-a systematic mapping review. submitted to Public health Nutrition (awaiting reviewer assignment). PROSPERO: International prospective register of systematic reviews. Unique Identification Number: CRD42015017749. 2015. (as part of MPH research attachment, 2014-15) - publication of a protocol for systematic reviews "Verstraeten R, Hawwash D, Lachat C, Bonn N, Pinxten W, Gillespie S, Holdsworth, Booth A. Nutrition prioritisation: three inter-dependent (mapping, methodology, and ethics) systematic review outputs. PROSPERO 2016:CRD42016043805. | **Interviews:**  Conceptual framework and process notes:   - *“we really need to look at what happens from the ground and look at how the stakeholders themselves see how it could be adapted or adopted. I don't know if it could be adopted in its entirety but, I think people see themselves at different places on that conceptual framework and maybe we can look at it and say ‘OK the systems that we are doing now [for decision-making] maybe it's not so OK, and they could be strengthened if we did this it way, [the way] that the EVIDENT framework is suggesting”* - *“maybe we should have Africanised EVIDENT, even from the very conceptual beginnings”* - *“I believe there’s also a nutrition initiative going on in South America at the moment. I think we could have perhaps learnt more from comparable frameworks and approaches. I don't know exactly what components they might have that we are missing, that would only come from examining their model, but I think we perhaps* missed an opportunity to identify that” - *“I think it is good to have a guideline [process notes]. Maybe there are so many guidelines [process notes] existing. But looking at those guidelines [process notes] and trying to come up with something adapted for EVIDENT specific point or EVIDENT scope it very important.”*   Shorter/quicker evidence synthesis products:   - *“I think that the choice to focus on the systematic reviews is understandable from an academic viewpoint, but systematic reviews are a longer path to implementation and so certainly when EVIDENT was conceived, I believed that there would be a more mixed portfolio of types of products and so I feel that perhaps in the pursuit of the longer term goals of developing skills in systematic reviews that may be the policy briefs side of things has may be taken a lesser role”* - *“I think there are still opportunities to do more rapid approaches to summarizing the evidence that may be capitalised more on narrow windows opportunity rather than longer standing problems”* |
|  | **Online Survey:**   - Survey responses indicated participants were most aware of the stakeholder mapping notes; they were the most used and thought to be the most helpful. Respondents were increasingly less aware of the other process notes; fewer used these notes, and fewer thought they were helpful. - 5 out of 8 African survey respondents found this step of engaging stakeholders to be ‘difficult’ or ‘very difficult’ (range from ‘very easy’ to ‘very difficult’). - The difficulty was further reflected in the extent to which stakeholders were actively engaged with EVIDENT; 5 out of 8 respondents said ‘somewhat’, ‘a little’ or ‘very little’ when measuring the extent of the active engagement. - The general consensus for carrying out the various processes of EIDM, namely obtaining prioritised research questions (5/5), contextualising systematic reviews (2/4) and generating evidence products (5/7) was that it was difficult. - Half of the survey respondents (4 out of 8) did not reach the stage of contextualisation, while 1/8, 3/8, and 1/8 did not identify and engage stakeholders, obtain prioritised research questions, or generate evidence products, respectively. |  |
|  | **Interviews:**  Conceptual framework:   - *“it has been developed by partners and has been evolved over, and it's been evolving over time”* - *“I think this [conceptual framework] is the best way to go. You work with a framework that guides you, and this is a very good framework that really guides the EVIDENT network”* - *“It [the conceptual framework] looks simple. Simplicity is good. You could talk about it in one slide and you could talk about it to stakeholders, talk about EVIDENT, what is EVIDENT.”*   Process notes:   - *“It is clear that, it is useful to have guidelines [process notes]”* - *“they [the process notes] are really good tools. I think they deserve to be out there, and [be] used. And this may be my [only negative] comment: ‘are they really disseminated and used widely?’ I think they were very carefully developed and a lot of care went into them but I think we should disseminate a little bit better”*   Case studies:   - *”it [case studies] forced us to think about a number of issues that are, through that is happening in the country. Yeah, I think it raised awareness also amongst our colleagues about a number of things, well, [on] the importance of the whole EIDM [process]”* - *“Through that [case study] process we have learnt that so far, so far we’ve learned that decision-making is not a straightforward process for many of those that have to make decisions. They have to make decisions with many constraints, sometimes they actually don’t have the information that they need because it doesn’t exist. But sometimes even when they plan based on evidence that exists, they don’t get a funding to implement that. Meanwhile, some funder is waiting on the touchline and saying ‘I have funding for you to do something I am interested in’. So it’s an interesting experience, because it makes you rethink your assumptions. The assumptions are that you do a survey, you find a problem, and you know what exists in other places for addressing it but you can’t prioritise that because you don’t have funding to deal with that, you don’t have local funding to deal with that.”*   Importance of stakeholder engagement:   - *“you can’t do something to succeed without engaging the relevant stakeholders”* - *“involving stakeholders during question development is very critical and also during prioritisation of the question or the research agenda is very critical. We appreciate I mean we have to involve them, otherwise you cannot ask them to use it [evidence] at the end. So the first thing is, it is important to get the right question from the stakeholder point of view they have to be agreed and you have to prioritise it together and then you have to start working so you have to keep informing them. So this is also very important”*   Difficulties/challenges in stakeholder engagement:   - *“the biggest difficulty is the first part: linking up with policymakers”* - *“that is often a difficult process because people are not very willing to share information and like we are busy they are also very busy so it’s hard to get a hold of them and to be able to fix interviews with them and complete it”* - *”evidence isn’t all that [that] is going into decision-making, and a lot of important decisions are made outside the evidence cycle, and that is extremely important to understand what those influences are. Because while evidence can yes inform decisions and that is what we want to happen, we can’t run away from the fact that there are also other influences out there that [can] influence decisions and we need to know how those work, because we need to be able to leverage both ends of the spectrum for decision-making”* - *“what I see is that often they [decision-makers] don’t have the freedom to make their own choices. Then you’ve got the World Bank coming [in] with millions of dollars saying ‘we would like to do this’ and then it’s not a question of ‘yes’ or ‘no’ but it’s a question of ‘do you want the money or not?’ and most people will say ‘ok, yes’ because this is money for the country. It’s a grant or whatever, so let’s do it.”* - *“Like in Ethiopia they are really strong about that [making their own decisions] now. They say ’look we want to decide what happens in our country’, up to certain degree but still, I mean. Now they want NGOs to be registered. They want a list of activities. They want to know what the NGOs are doing in their countries. But that is not mainstream. In many countries that’s not the case.”* - *“what EVIDENT is trying to do is quite new to them. It is not common that researchers and stakeholders sit together and discuss a question that would be studied by the researcher and then the results are given back to the stakeholders. I mean there is no continuous flow of information between the 2 categories. So this might not be new to the world but at least in Benin this was new thinking, a new way of doing things. Therefore people were – I won’t say doubting – but they were not eager to participate. We had to convince them that this was useful and it was difficult to convince them in that.*” - *“you don’t get to the top of their agenda simply because they don’t know you. But if they do know you and you do have some sort of reputation, then they do actively listen to you. You know, to your request”*   Difficulties to EIDM overall:   - *“It’s more confirmation of the fact that evidence isn’t all that is going into decision-making and that a lot of important decisions are made outside the evidence cycle, and that is extremely important to understand what those influences are because while evidence ‘yes’ can inform decisions and that is what we want to happen, we can’t run away from the fact that there are also other influences out there that influence evidence[-based] decision[-making] and we need to know how those work, because we need to be able to leverage both ends of the spectrum for decision-making. So that would be the lesson for me so far.”* - *“it made me aware of the importance of re-contextualising the whole idea from an African context”* - *“an economic evaluation. Mostly we just deliver services without knowing which costs we are hankering. Whatever costs we are gaining, we don’t know really. I think it is quite difficult to say we really 100% consider the economic aspect of our intervention. So in that regard we also require a lot of expertise. There is also a shortage on expertise of like health economists. So involving those kinds of expertise is very important, besides having a nutrition background. Most of us are, we are from nutrition background, but we also require health economists who can help us to whatever intervention, whatever effective intervention, whatever alternative we came up, what is the cost and how we consider it from an economic point of view”* - *“Because a systematic review in itself is taking a long long time. It’s 6 to 12 months, so that is too much I think.”* - *“EVIDENT was never anyone’s main job. It was always something that people thought ‘yes, this is interesting. I want to get involved with it’”* - *“challenges that have been faced by the different country groups. The capacity related challenges and stuff like [that]. And I have said this to [the coordinator] several times when I say this [EVIDENT] is a capacity strengthening activity.”* - *”there was some confusion about the goals [of EVIDENT]. I suppose that just because we were developing what EVIDENT was, it’s a new kind of partnership really, it's not a research project which most of the academic partners are used to, where we can identify what our aims are, what our outputs would be, so [that] we can justify our involvement in our own institutions. So yeah I think there was some confusion there”* - *“time is short and the expectations are great”* - *“we are amateurs at this. We're new to this, and so I think that it’s [been] a little bit, how should I say it, ambitious, you know within a year, for us it’s been within a year, year and a half to accomplish of these things”* - *“it all comes down to our own commitment to achieving these outputs. So we have to prioritise them and if we don’t get to it, it means we didn’t prioritise it high enough”* - *And so we tried to do something in the south where we actually are [sitting] in the north and the context is actually in the south. So you take it out [of the northern context] and then you bring it back in [to the south] which doesn't, I don't know if I make myself understandable but I think that the big weakness.”*   Barriers to POEIDM on the ground:   - *“I don’t think that many policymakers are used to start thinking and making their own decision. It’s just not in their culture. But it’s also something that they haven’t learned to do.”* - *“you bring a whole bunch of people together from multi-disciplinary backgrounds and so how you manage that when you do a systematic review, that's what I'm actually talking about, because you have to work with different people at different times, different countries, different cultures, different ways of performing to work”* - *“there is a lot of bureaucracy, you can't just approach people by word of mouth or even by email, people want formal letters because they want a paper trail to be on file that people contacted us and I spoke to these people and everything. So we had to do formal letters, follow up with e-mails and phone calls** to be able to get those appointments.”* - *“Experience in real time, a very disruptive power cut which lasts for four hours all of a sudden gives you this whole new insight into why sometimes things take a whole lot longer in some countries”* - *“We sometimes also have a limitation regarding internet. My internet is down, all the time down. Or it is slow. When it [is] slow, you are disappointed.”* - *“[we] don’t have access to let’s say Cochrane, [and] this [is a] very very accurate database”* |  |
| **Horizontal Collaboration** | | |
| **Network & Communication** | **Documentation:**   - A kick-off meeting was organised for EVIDENT partners, as per the protocol. This meeting was held in January 2014, with all EVIDENT partners in attendance. The protocol also specified a follow-up meeting in mid-2015. EVIDENT organised a total of 5 follow-up meetings with all of its partners. - Smaller face-to-face meetings, email, Skype, Cisco WebEx, WhatsApp, phone calls and the project website ([www.evident-network.org](http://www.evident-network.org)) facilitated continuous communication. | **Interviews:**  Regular updates, more face-to-face contact:   - *“There is barely any periodic get together to see how things are going, where things are going. I think that it would be nice to have more opportunities to interact also a bit”* - *“I think we had opportunities to meet, but it wasn’t enough. I think that EVIDENT should have structured a lot more contact sessions between country teams. I think we have a lot to learn from each other.”*   Diverse communication streams:   - *“We should diversify the tools of communication, tools we are using. Relying on a website that has just been updated once, it is not sufficient. They should do much more, workshops, conferences, other tools. I hear that EVIDENT is also organising a conference in November so these kinds of tools are useful for communication. It’s a good communication strategy but it’s not enough, also look at newspapers, scientific conferences like the one which is held in Morocco right now, the ANEC and others, maybe writing books, writing articles, scientific academic but also in practitioners’ reviews and grey literature. All those yes, could be useful. So diversify methods of communication would be my guess”*   Coaches teaching role:   - *“Perhaps in hindsight, we could have involved some of the other coaches in the delivery of the initial course and then that would have made relationship building a bit easier”* - *“EVIDENT should create teams to create partnership that allow to work on the same topic but with different expert within countries but also across countries, that we have people more useful because I felt like an ivory tower while working on my systematic review”*   Buy-in:   - *“There’s been partners who’ve been highly active and then there’s been the partners who’ve been less active. I think there’s even some partners that we keep on there because the name is really good to have but the contribution is almost zero. So then if I would give a suggestion on how it can be improved I really think there should then be buy-in from all the different collaborators.”* - *“the lesson is to have a good team, good staff, fully assigned to the activities and to pay them for that”*   Link EVIDENT with existing organisations/networks/platforms:   - *“link with an existing platform instead of creating a new platform which is something very difficult and very challenging for every country [to achieve]. Trying to link with an existing platform is very important. A platform that [already] involves researchers [and] academia that are generating evidence”* - *“We should communicate with all the stakeholders efficiently, in terms of resource mobilisation, in terms of collaboration. I think there should be…much [more] should be done especially linking those international partners like IFPRI, globally important stake on the nutrition agenda.”* - *“we need to work with at the country level others partners, those key partners like those UN agencies... If they are part of the local working group it would good in resource mobilisation”*   Outputs/evidence products for dissemination:   - *“initially I think the communication was about selling promises and selling potential. And in a month’s time we will probably be able to sell track record. And say ‘this is what we have done. You know at this, this is one of the outputs’ so then it becomes probably a bit easier”*   Hire more staff/personnel:   - *“So there is some communication but not enough I mean. It is not enough. And it may not be also clear and understandable by every EVIDENT group member. I don’t think it’s something intentional. But because of the workload and lots of several competing interests the communication is not really going as we require or as we needed. That is why I also suggest someone fulltime, especially for communication you know: communicating and coordinating and trying to reaching EVIDENT group and creating awareness and creating a platform for communication and also linking with other partners. It really requires a lot of effort. Now everybody is putting some portion of time, which is somehow ok but really communicating and coordinating is a big challenge”* |
|  | **Online survey:**   - With the exception of just one (‘neither satisfied nor dissatisfied’ and ‘average’), all survey participants were satisfied with their work in EVIDENT (13/16 ‘satisfied’, 2/16 ‘very satisfied’), and felt that their experience of working within EVIDENT has been good (13/16 ‘good, 2/16 ‘very good). - 15 out of 16 felt at ease to discuss issues within the group - All (16 out of 16) survey respondents felt they were listened to when they spoke up - All respondents were satisfied with the communication at face-to-face partner meetings (all responses were ‘neither dissatisfied nor satisfied’, ‘satisfied’ or ‘very satisfied’). Respondents were also satisfied with the in-person communication during trainings (15/16) and at conferences (11/15). - Online surveys revealed that on a scale of ‘never’ to ‘very often’ writing (5/16 ‘very often’) was the most common form of communication amongst colleagues, followed by speaking to each other (2/16 ‘very often’). Meeting up was the most infrequent form of communication used (1/16 ‘very often’). - Email was less popular as a communication medium amongst the participants as only 7 out of the 16 were ‘satisfied’ or ‘very satisfied’. - EVIDENT website was the least popular media, with only 4 respondents satisfied with it. 10 out of 16 said they were ‘neither dissatisfied nor satisfied’ with the website, while 1 was ‘dissatisfied’ and another was ‘very dissatisfied’. - 6 out of 8 survey respondents felt that they were only ‘somewhat’, ‘very little’ or ‘not at all’ aware of the activities going on in other country teams. |  |
|  | **Interviews:**  Functional network, existing relationships, shared values:   - *“I think it was very functional so we had good communication with [the coordinator] and the other members of the ITM part of the network. We had good internal communication, so [amongst the Sheffield team] and certainly when [the coordinator] and or other members of [the coordinating body] came to visit or we visited them, it was all very functional”* - *“I mean it was very functional. As I said, I mean, some of these collaborations were already existing.”* - *“I think we have a compatibility in value systems between the ITM group and ourselves. And that for us is really important. It’s a really important place to start off with, because if you don’t have that compatibilities in values, then it’s a non-starter”*   Networking opportunities (to work together, share knowledge, connect and support each other, build friendships):   - *“when you are trying to do something as an individual and now you have a group that is well-organised to do this, it is better to just join them than do individual work”* - *“I think also the advantage is that you can have different people at different levels of expertise or capacity and these can be exchanged and you can learn from one another and that's what makes it interesting and dynamic to be part of such a network. You can always learn something from somebody else.”* - *“I think one of our best moves has been to start collaborating with IFPRI gave us the chance to go to LCIRAH to the academy in Ethiopia, you start talking to people, you talk to A who talks to B and then you go to C and so sort of in the international environment right now, people know what/who EVIDENT is, I think also the collaboration with SUN at FANUS conference was quite constructive.”* - *“there are very few opportunities like this for people within the global south and north to work together, and as I said I see EVIDENT as the way to be able to bring the researchers in the global south and north to work together”* - *“there’s also the friendships that have evolved”* - *“it’s very valuable because there is opportunities to elaborate across our boundaries, like pull us together, publish together etc and we have met others that maybe who not normally find ourselves relating to in terms of the other networks, IFPRI etc, so it's been good.”*   Credibility & visibility:   - *“we had ITM as a partner and ITM is based in Europe, so it gives you a better standing to say ‘we are also engaged with ITM’, and that means a lot when we talk about north and south collaboration. It brings a certain credibility to…an African university working with a partner in Europe and working with European funds”* - *“So for us, for research, it’s very valuable because there are opportunities [within EVIDENT] to elaborate across our boundaries, like [those that] pulls us together, publish together etc”*   Imbalance in interactions (coordinating body, case study teams, external stakeholders):   - *“it will still be difficult to get the balance right between who’s taking the lead because in the same sense I mean I think from [the coordinating body], many many times it has been said ‘this should really be a south driven project’ but you realise it never was a south driven project from the south.”* - *“EVIDENT is a south-south, south-north corporation, but it’s much more led by the north”* - *“I am talking of these funding agencies, the SUN Movement, which I don’t know how much communication has been going on with these organizations”* - *“if you want to talk to funders and donors, everybody is in the north and so it's much more convenient for us [the coordinating body] to talk to these people than it is for people in the south. But I still think that that's a big weakness because you can't bring people over to just have a face to face talk. It's expensive, its whatever so it's always a little bit. I think that's been, it's something very important and something we need to take into account that if you want to talk to the big players they're all in the north but if you're actually living and working in the south, they just feel sometimes so disconnected of what is being said in the north”*   Communication streams/media:   - *“I really like the fact that there were a number of face-to-face interactions, with these country meetings. And I think they are really important. And if it had not been for those face-to-face interactions, I think EVIDENT would not have worked at all”* - *“weaknesses related to the sort of intensity of involvement when we have face-to-face meetings and the dissolution of that impetus when we separated. So it was very hard to keep the momentum going”* - *“Again I think email is probably the worst way of communicating. And sending emails and assuming that people will read them, I think is not the most effective one. But it’s practical, it’s cheap to do and so on and so on and so on”* - *“EVIDENT is forced, they’re forced to make internet-based communication and so we all know that in Africa that’s not always possible”* - *“for instance we had Skype calls, even if it is planned [on] a [specific] day, some people could not attend just because the internet is not working at that moment [in time]”* - *“Relying on a website that has just been updated once, it is not sufficient”*   Challenges with communication:   - *“I think it was just difficult for people to commit to people they have never seen or never met, who were 10,000k away. I think that was a real problem”* - *“I would be able to help with suggestions if I knew what we are trying to deliver on. I’m not still sure what we’re trying to deliver on”* - *“the interaction between different people made it a bit difficult…I mean you’ve got people that don’t [know] each other. You put them together and that’s not easy. Some of them [have] had contact only through email; they never saw the person who was their coach. It’s difficult.”*   Imbalance in interactions (coaches-case study teams):   - *“if it [EVIDENT] didn’t function it was not due to the structure itself, it was due to the relationships and the fact that some people were too shy to ask questions to their coaches or they were not specific enough to their coaches and then the coaches were not happy”*   *“but it was not always that fantastic guidance …We would say something and maybe three weeks later we would get feedback. But when your counterparts want feedback already a week later… I think that kind of technical support to me is what was missing”*   - *“I don't know whether there were tangible south-south collaborations. I mean they're obviously south-south discussions that happen in the[face-to-face] meetings, comparing experiences and learning but I don't know whether there were any examples of direct collaboration”* - *“the communication between the [case country] teams has been quite difficult but now it is changing a bit”* - *“I don't know what's happening locally in other [case] countries”* - *“the trust takes a long time, [especially] building good relationships with your partners across countries. I've seen [that], and that's like a personal [side] note that it's sometimes much more difficult for African partners to work with one another and have faith in one another than it is for them to work with people in the north”* - *“it’s difficult to connect at the different level of for instance Ethiopia and Benin kind of started it seems like in a different way than South Africa started and so sometimes when we're having discussions there's this, how should I say we don't see eye to eye**because we’re at different places”* |  |
| **Visibility** | **Documentation:**   - EVIDENT developed an operational and user-friendly website ([www.evident-network.org](http://www.evident-network.org)) as intended; the website had amassed 7336 views by 20/12/2016. - EVIDENT was showcased at 12 international conferences over the period of 3 years. No conference was originally planned.   List of conferences:  SUN Movement Global Gathering – Rome, Italy – November 16-18 2014; World Congress of Public Health Nutrition II– Spain – November 2014; 3rd Federation of African Nutrition Societies Conference - Arusha, Tanzania - 27-28/5/2015; SUN Movement Global Gathering – Milan, Italy – October 20-22 2015; LCIRAH- Agriculture, Nutrition and Health Academy - Addis Ababa, Ethiopia - 20-24/06/2016; World Public Health Nutrition Association’s World Nutrition Congress – Cape Town – 30 Aug-2 Sep 2016; ANEC - Pretoria, SA - 20-22 September 2016; African Nutrition Epidemiology Conference VII - Marrakesh, Morocco - 9-14/10/2016; Regional Strategic Analysis and Knowledge Support System Conference - Accra, Ghana - October 18-20 2016; Micronutrient Forum Global Conference - Cancun, Mexico - October 24-28 2016; 2^nd^ World Breastfeeding Conference - Johannesburg, South Africa – 11-14 December 2016; International Congress of Nutrition - Buenos Aires, Argentina - 15-20 October 2017. | **Interviews:**  Linking EVIDENT with organisations/networks:   - *“EVIDENT should partner with a much bigger and a more visible group, such as either PRICELESS and Wits University or with the Centre of Excellence of Food Security, because they report back to the government. They are institutionalised, whereas our project at North-West University is just between us and EVIDENT and doesn’t tie back to the government. I am just talking from the South African perspective”*   Strengthening existing relationships:   - *“EVIDENT continues its very favourable relationship with the SUN because the SUN Movement can get things…well can get attention, I was going to say get things done but it’s mainly the attention, they can command attention around the world”* - *“IFPRI is active on the ground in many countries especially through the agriculture, nutrition agriculture relationships. That’s also another place that we could get some good traction in getting evidence...in the evidence that we generate and the evidence products directly applied. So, funding and good friends I guess“*   New relations:   - *“maybe more involving as much as possible more countries, because at the moment we have around 6 countries of Africa. Like Tanzania, Ethiopia, Ghana, Benin and SA and Morocco. Having more countries it will give also a lot of visibility.”*   Local in-country visibility:   - *“the EVIDENT groups within country should organise a little bit of workshops and seminars, and invite policymakers to take part in these processes. This is the only way you can make yourself visible in the country, you know”*   Funding**:**   - *“I think it takes money. You need to organise meetings, you need to travel, you need to pay staff to…it costs money to be out there. I think it’s about funding. I think the procedures were there. The groundwork was there but it required much more money”* |
|  | **Online survey:**   - EVIDENT’s attempts at increasing visibility were perceived to have worked ‘fairly well’ or ‘extremely well’ by 75% (12 out of 16) of the survey participants. |  |
|  | **Interviews:**  Successful overall:   - *“it's been good visibility, people have heard of it they know of its [EVIDENT’s] mission and goals”* - *“yeah I think they [the coordinating body] did a good job of promoting the project internationally, really good job of getting it [EVIDENT] known”*   Co-ordinating body role:   - *“I think regarding visibility we've [the coordinating body] done our best considering our links with SUN, with IFPRI, the word of mouth and how it worked, to go to conferences, to be visible to be present and I think that has been very efficient.”* - *“even before I became part of the EVIDENT Movement…became part of EVIDENT I was in a SUN Movement meeting in Rome where [the coordinating body] was playing a leading role in issues related to how evidence is used for decision-making”*     Little funding:   - *“I think it takes money. You need to organise meetings, you need to travel, you need to pay staff to…it costs money to be out there. I think it’s about funding. I think the procedures were there. The groundwork was there but it required much more money”* |  |
| **Sustainability** | | |
| **Sustainability** | **Documentation:**   - Protocol requirements were met when EVIDENT succeeded at securing funding through Agriculture for Nutrition and Health (Supporting Policies, Programs, and Enabling Action through Research ) at IFPRI. EVIDENT will use this to write a protocol for large scale funding. | **Interviews:**  Continuation of capacity building:   - *“we can go ahead and do training and build the capacity. We can incorporate some of these things into [our] curricula, you know in our training institutions, doing [the] systematic [reviews]. You know over there to produce systematic reviews, our students don’t do systematic reviews but this could be something you know [that] we need to begin to think about curricula so that the capacity is there [locally]. We need a critical mass to keep this thing going and so that’s definitely one of the things that needs to be done; it needs to go beyond the network, it needs to go beyond institutions”*   Contextualise EVIDENT:   - *“we might have missed an opportunity to re-contextualise the idea from an African perspective”* - *“maybe we should have Africanised EVIDENT, even from the very conceptual beginnings”*   Increase ownership:   - *“[what] we saw is that right from the start there was a very clear and avert, let’s call it intention from the northern partners that the ownership should be in the south. And I don’t think that ever happened. I think the southern partners…I think we simply…sometimes I think we don’t know how to do it. But sometimes I also think that we are so used to – this sounds like an excuse – being dictated to, that these things come along and we just sit back and relax and wait. and I think that, yeah, that was a bit worrying.”* - *“there was a decision for North-West University and the University of Ghana to come on board and lead the process for the African team. Which I thought was a very good thing if it is adopted because then it creates a strong ownership on the continent, an ownership aspect on the continent.”*   Set new goals and objectives:   - *”I think yes we all want EVIDENT to continue and to succeed but there is no purpose in something like that if it’s just for the purpose of continuing. Actually new goals should be set and new objectives”* |
|  | **Online surveys:**   - Almost all participants perceived that EVIDENT had not become sufficiently sustainable. Only 1 survey respondent felt that the sustainability of EVIDENT was fine as it was. Meanwhile, 50% (8/16) thought EVIDENT needs some work to improve its sustainability, while 56% (9/16) thought EVIDENT needed major work. |  |
|  | **Interviews:**  Reduce dependency:   - *“I think this project was, it was coordinated by us [ITM], we strengthened, we provided trainings, we strengthened the skills of our partners and so the two of them came forward saying, together we will have sufficient leadership capacity to take this up.“* - *“a partner said, “But if we're going to do that [tender health technology assessments] then we are never going to be able to compete with the others because we just don't have the expertise” and it again will be people from the north, who will receive all benefits and so it's not fair, and that's where it changed and [we] said [that] then we will focus on training and give you [the south] capacity and hopefully the leadership to actually do it or train your own people in your country because that's been always the purpose trying to, which is why we held the training”* - *“there was a decision for North-West University and the University of Ghana to come on board and lead the process for the African team. Which I thought was a very good thing if it is adopted because then it creates a strong ownership on the continent, an ownership aspect on the continent”*   Funding:   - *“sustainability is really linked to dedicated funding”* - *“we are more or less at crossroads where without any such funding then EVIDENT would crash land so [we] have to actively pursue the process of looking for funding for the next phase, that shows how critical it is. Without funding we won’t be able to make any progress”*   Need outputs/evidence synthesis products:   - *“all the funders wanted outputs first of all, which is quite a logical and second, you need to be able to show what you've done and evaluate what you've done before you can actually get additional funds.”* - *“if we are able to you know achieve some success and demonstrate that look EVIDENT has done this and this is [it] what has [done], I mean to show that the existence of this network has resulted into this, then I don’t see why EVIDENT should not exist”* |  |
| Key: ANEC: Africa Nutritional Epidemiology Conference; EIDM: evidence-informed decision-making EVIDENT: Evidence-informed Decision-making in Nutrition and Health; FANUS: IFPRI: International Food Policy Research Institute; ITM: Institute of Tropical Medicine; LCIRAH: Leverhulme Centre for Integrative Research on Agriculture and Health; NGOs: non-governmental organisations; PRICELESS: Priority Cost Effective Lessons for System Strengthening; SUN: Scaling Up Nutrition; UN: United Nations; … = short silence, ** = laughing | | |
